# Morphological, biochemical, transcriptional and epigenetic responses to fasting and refeeding in intestine of *Xenopus laevis*

**DOI:** 10.1186/s13578-016-0067-9

**Published:** 2016-01-21

**Authors:** Keiji Tamaoki, Reiko Okada, Akinori Ishihara, Nobuyoshi Shiojiri, Kazuki Mochizuki, Toshinao Goda, Kiyoshi Yamauchi

**Affiliations:** Department of Biological Science, Graduate School of Science, Shizuoka University, Shizuoka, 422-8529 Japan; Green Biology Research Division, Research Institute of Green Science and Technology, Shizuoka University, Shizuoka, 422-8529 Japan; Department of Local Produce and Food Sciences, Faculty of Life and Environmental Sciences, University of Yamanashi, Kofu, 400-8510 Japan; Laboratory of Nutritional Physiology, School of Food and Nutritional Sciences, The University of Shizuoka, Shizuoka, 422-8526 Japan

**Keywords:** Fasting, Refeeding, Intestine, Metabolism, Transcription, Epigenetics, *Xenopus laevis*

## Abstract

**Background:**

Amphibians are able to survive for several months without food. However, it is unclear what molecular mechanisms underlie their survival. To characterize the intestinal responses to fasting and refeeding, we investigated morphological, biochemical, transcriptional and epigenetic changes in the intestine from adult male *Xenopus laevis*.

**Results:**

Frogs were fed for 22 days, fasted for 22 days, or fasted for 21 days and refed for 1 day. Fasting reduced, and refeeding recovered partially or fully, morphological parameters (wet weight of the intestine, circumference of the epithelial layer and number of troughs in a villus-trough unit), activities of digestive enzymes and plasma biochemical parameters (glucose, triglycerides, cholesterol and free fatty acids). Reverse transcription-quantitative polymerase chain reaction analysis revealed overall suppression of the transcript levels by fasting, with various recovery rates on refeeding. Chromatin immunoprecipitation assays on the selected genes whose transcript levels declined with fasting and recovered quickly with refeeding, showed several euchromatin marks in histone (acetylation and methylation) and RNA polymerase II modifications (phosphorylation) with fasting, and returned to the feeding levels by refeeding. The mRNA levels of these genes responded to fasting and refeeding to greater extents than did the pre-mRNA levels, suggesting the involvement of post-transcriptional regulation.

**Conclusions:**

Our results demonstrate that the *X. laevis* intestine may undergo overall metabolic suppression at least at the transcriptional level to save energy during fasting and quickly recovered to moderate nutritional deficiency by refeeding, and suggest that these dietary responses of the intestine are epigenetically and post-transcriptionally regulated.

**Electronic supplementary material:**

The online version of this article (doi:10.1186/s13578-016-0067-9) contains supplementary material, which is available to authorized users.

## Background

The intestine is a dynamic organ that adjusts to changing energy demands and supply. At the cellular level, small intestinal epithelial cells have a strictly controlled mechanism depending on a balance between cell proliferation and death. In mammals, small intestinal epithelial cells have a high turnover, renewing every 4–5 days [1]. Stem cells proliferate in the crypt base and rapidly dividing cells migrate upward to the villus tips of the intestine, where terminally differentiated cells undergo apoptosis [1]. Refeeding after a period of short fasting stimulates cell proliferation and migration, and reduces apoptosis with intestinal structure and functions recovering within 3 days [2]. In rats, fasting for more than 1 week causes gastric mucosa disorders [3], such as atrophy of intestinal structure and functions, resulting in bacterial translocation [4]. In spite of a large body of information describing the effects of food deprivation on intestinal structure and functions derived from findings in studies on short-term fasting in endothermic laboratory animals and humans, it is unclear what molecular mechanisms underlie gastric mucosa disorders or if these pathological conditions can be prevented.

Intestinal flexibility is highly adaptive in response to feeding and fasting, and the frequency of feeding in nature differs among vertebrate species. Unlike vertebrate endotherms whose digestive tract is rarely empty in usual conditions, vertebrate ectotherms can live for relatively extended periods without food [5], e.g., during hibernation in winter season and aestivation in dry summer season, when they spawn and rear offspring, and when they encounter with prey by chance. Some amphibians can survive for several years in dry conditions without food [6]. Although amphibians have resting metabolic rates that are less than those of endotherms by at least one order of magnitude [7], the long-term survival of vertebrate ectotherms without food cannot be explained by their low metabolic rates alone. Indeed, the plasticity in the intestinal structure and function of vertebrate ectotherms is higher than that of vertebrate endotherms. In addition, adaptation mechanisms, such as the down-regulation of resting metabolic rate during fasting [8], so-called metabolic rate depression [9], are present in vertebrate ectotherms. Secor [10] proposed that the high plasticity in the intestines of vertebrate ectotherms may involve the remodeling of the structure and/or function in the intestinal epithelial cells. Understanding the cellular mechanisms responsible for the intestinal plasticity in vertebrate ectotherms may provide novel insights into the pathogenic mechanisms of severe intestinal atrophies found in rodents and humans during prolonged fasting.

This study was conducted to examine the effects of fasting and refeeding on the morphological, biochemical, transcriptional and epigenetic responses involved in intestinal functions of *Xenopus laevis*. Frogs were fed ad libitum for 22 days, fasted for 22 days, or fasted for 21 days and refed for 1 day. We investigated the structure of the intestines, the activities of digestive enzymes, the plasma concentrations of glucose and lipids, the intestinal transcript levels by reverse transcription-quantitative polymerase chain reaction (RT-qPCR) and epigenetic marks of selected genes by chromatin immunoprecipitation (ChIP) assay.

## Results

### Intestinal morphology and plasma biochemical parameters

**Fig. 1:**
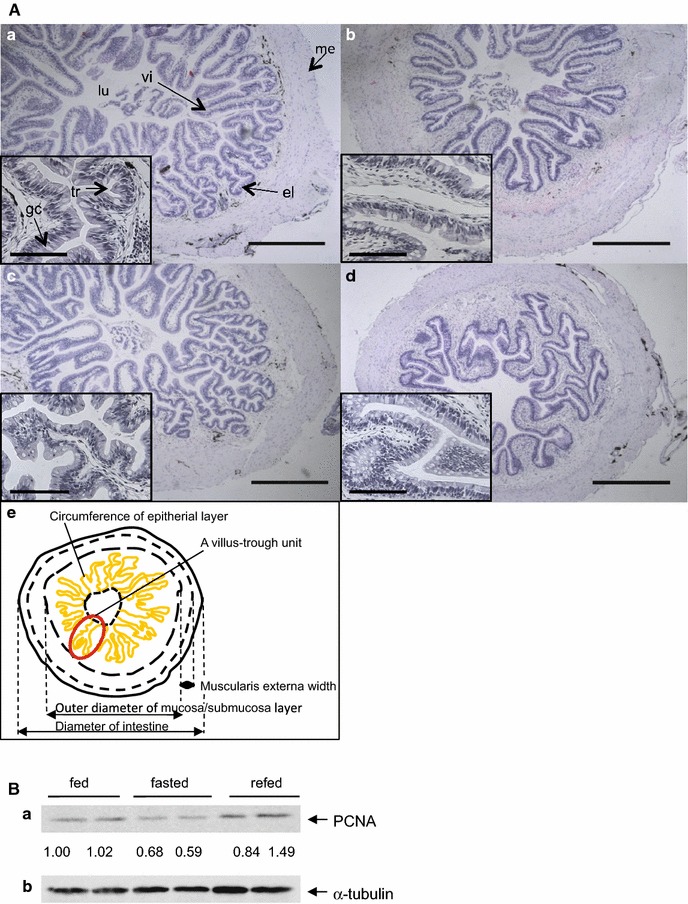
Morphology and a cell proliferation marker in the intestines of fed, fasted, and refed *X. laevis*. **A** Histology of representative intestines of fed, fasted, and refed *X*. *laevis* stained with hematoxylin and eosin. The frogs were fed for 22 days (*a*), fasted for 22 days (*b*), or fasted for 21 days and then refed for 1 day (*c*), and fasting for 5 months (*d*). *e* Schematically illustrated structure of the *X. laevis* intestine with morphological parameters measured (see Table 1). The *box* in each *panels* (*a*–*d*) is enlarged image. *el* epitherial layer, *gc* goblet cell, *lu* lumen, *me* muscularis externa, *tr* trough, *vi* villus. *Bar* 500 μm in *panels a*, *b*, *c* and *d*, and 100 μm in *boxes*. These experiments were repeated at least three times, with similar results. **B** Protein expression of proliferating cell nuclear antigen (PCNA) in the intestines from fed, fasted, and refed *X*. *laevis.* Intestine homogenates (60 μg protein; two samples/each group) were analyzed by SDS-PAGE, followed by Western blotting. Band intensities were analyzed and expressed relative to α-tubulin, and values are expressed relative to the value of the fed frog (*left*) that was set to 1.00. (*a*) PCNA; (*b*) α-tubulin

During the course of experiments, no significant changes were observed in body weight within every group. There were also no significant differences in body weight, intestine length and diameter, outer diameter of the mucosa/submucosa layer, muscularis externa width, number of the goblet cells, and protein content per tissue on Day 22 among the three groups (Table 1). The wet weight of the intestines was significantly smaller in the fasted frogs (52 %) than in the fed frogs, and recovered partially on refeeding. Unlike the villus-crypt structure in rodent and human intestines, the villus-trough in the frog intestine had a complicated structure with highly branched troughs (panels a to e in Fig. 1A), therefore we could not simply compare the villus length among the three groups. The troughs branched in the fasted frogs to a lesser extent than in the fed and refed frogs: the numbers of troughs in a villus-trough unit was ~2.7 versus 4.6–8.0, resulting in a decrease in the circumference of the epithelial layer to 57 % of the fed frogs. Although almost all scores of these morphological parameters were slightly smaller in the frogs fasted for 5 months than in the frogs fasted for 3 weeks, there were no striking differences between them (panel d versus panel b in Fig. 1A). The integrity of the intestinal epithelial layer looked similar in all frogs of the three groups at Day 22. Even in the intestine from frogs fasted for 5 months (panel d in Fig. 1A), we could observe no impairment of the intestinal structure. Digesta was still present in the rectum of all frogs even after 5 months of fasting. Immunoblot analysis indicated that the amounts of proliferating cell nuclear antigen (PCNA) in the intestine homogenates from the fasted frogs were less than those from the fed frogs, and that the amounts in the refed frogs recovered to the levels of the fed frogs (Fig. 1B). The histological staining of intestinal alkaline phosphatase activity revealed that a positive signal was localized in the brush border membranes of the intestinal epithelial layer, and that the signal intensity was lower with fasting than with feeding and recovered partially with refeeding (Additional file 1: Figure S1).

**Table 1:** Morphological changes of small intestines and biochemical changes of plasma parameters in fed, fasted and refed *Xenopus laevis*

	Fed group	Fasted group		Refed group
	22 days	22 days	5 months	1 day after
				21 days fasting
Sample number of group (*n*)	8	8	6	8
Body weight (g)				
Start of experiment	51.10 ± 2.74	51.39 ± 1.32	53.75 ± 3.01	52.69 ± 1.33
End of experiment	55.03 ± 3.05	48.30 ± 1.13	45.75 ± 3.10	49.65 ± 1.94
Intestine				
Wet weight (g)	0.54 ± 0.06^a^	0.28 ± 0.01^b^	0.22 ± 0.01	0.41 ± 0.03^c^
Length (cm)	5.50 ± 0.34	4.80 ± 0.29	5.05 ± 0.38	5.21 ± 0.32
Diameter (mm)	2.64 ± 0.27	2.21 ± 0.12	1.87 ± 0.07	2.40 ± 0.14
Outer diameter of mucosa/submucosa layer (mm)	2.26 ± 0.29	1.76 ± 0.10	1.45 ± 0.08	2.01 ± 0.09
Circumference of epitherial layer (mm)	35.01 ± 4.26^a^	19.83 ± 1.67^b^	13.88 ± 2.18	24.52 ± 4.22^a, b^
Muscularis externa width (mm)	0.19 ± 0.02	0.22 ± 0.02	0.21 ± 0.01	0.20 ± 0.03
Number of goblet cells/mm	68.12 ± 4.21	55.06 ± 13.42	48.2 ± 3.95	49.15 ± 9.69
Protein content (mg/g wet whight)	114.5 ± 7.02	102.8 ± 9.88	Not determined	99.41 ± 8.16
Number of throughs in a villus-trough unit	4.64 ± 0.88^a^	2.77 ± 0.43^a^	2.71 ± 0.19	7.97 ± 1.43^b^
Total troughs/section	62.25 ± 6.23^a^	30.42 ± 5.55^b^	26.11 ± 2.68	80.96 ± 7.45^a^
Blood				
Glucose (mg/dL)	44.55 ± 4.08^a^	28.38 ± 5.35^b^	41.52 ± 3.71	31.02 ± 3.25^b^
Triglyceride (mg/dL)	242.19 ± 51.82^a^	107.45 ± 14.30^b^	65.33 ± 16.15	141.00 ± 18.89^a, b^
Cholesterol (mg/dL)	173.97 ± 27.99	120.57 ± 16.44	141.00 ± 26.91	122.00 ± 13.80
Free fatty acid (mEq/L)	0.93 ± 0.08^a^	0.49 ± 0.05^b^	0.35 ± 0.06	0.62 ± 0.12^b^

Values presented are mean ± SEM (*n* = 8). Different letters (a, b and c) indicate significant differences between the groups (*p* < 0.05)

The data of the fasted group for 5 months are excluded from the statistical analysis

The concentrations of glucose, triglycerides and free fatty acids in plasma with fasting at Day 22 declined significantly to 44–64 % of those with feeding (Table 1). Although the cholesterol concentration of the fasted frogs was 69 % of that of the fed frogs, this decline was not significant. The concentrations of these biochemical parameters in the refed frogs at Day 22 were still low but had recovered slightly to 58–70 % of those of the fed frogs. In plasma of the frogs fasted for 5 months, the concentrations of these biochemical parameters except for that of glucose were comparable with those of the frogs fasted for 22 days (Table 1).

**Fig. 2:**
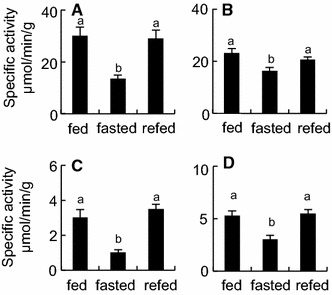
The enzyme activities in the intestines of fed, fasted and refed *X*. *laevi*s. Enzyme assays were conducted using the extracts obtained from the intestines of the frogs that were fed for 22 days (*fed)*, fasted for 22 days (*fasted*), or fasted for 21 days and then refed for 1 day (*refed*). The activities were expressed as μmol hydrolysed substrates or generated products/min/g protein. **A** alkaline phosphatase; **B** aminopeptidase; **C** glucoamylase; **D** maltase. *Values* presented are mean ± SEM (*n* = 8). *Different letters* indicate significant differences between groups (*p* < 0.05). These experiments were repeated at least three times, with similar results

The activities of intestinal alkaline phosphatase, aminopeptidase, glucoamylase and maltase with fasting at Day 22 decreased to 45, 70, 34 and 58 %, respectively, of those of the fed frogs (Fig. 2). However, the activity of these enzymes in the refed frogs had returned to those of the fed frogs within 1 day after refeeding.

### Transcriptional changes in intestine

**Fig. 3:**
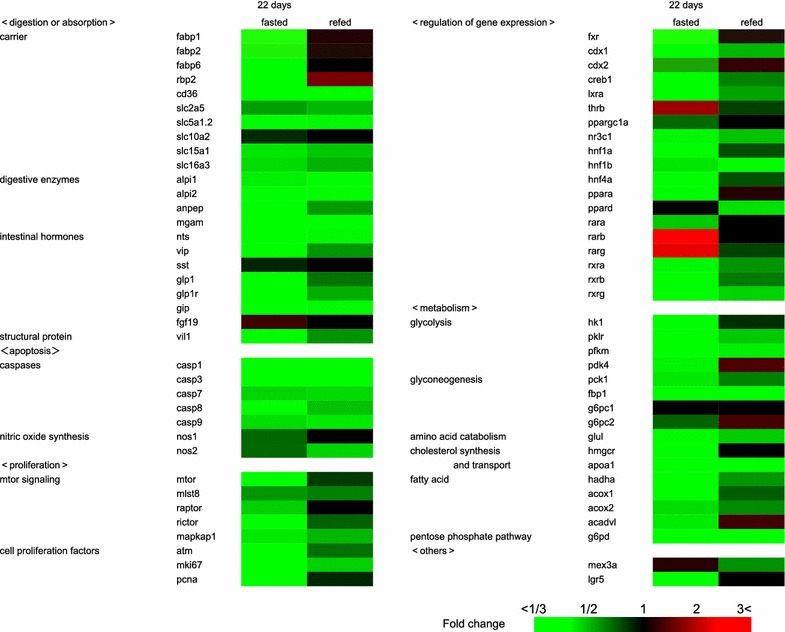
Reverse transcription-quantitative polymerase chain reaction (RT-qPCR) analysis of gene transcripts in the intestines of fed, fasted and refed *X*. *laevi*s. RNAs were prepared from the intestines of the frogs that were fed for 22 days, fasted for 22 days (*fasted*), or fasted for 21 days and then refed for 1 day (*refed*), followed by RT-qPCR (*n* = 8). Transcripts of 74 genes were divided to the following categories: digestion or absorption (22 genes), apoptosis (7 genes), proliferation (8 genes), regulation of gene expression (19 genes), metabolism (16 genes) and others (2 genes). *Color scale* indicated fold changes of gene expression. The data of the full name of the genes tested, the exact fold changes, SEM and statistic analysis are shown in Additional file 2: Table S1. These experiments were repeated at least two times, with similar results

The effects of the fasting and refeeding on the amounts of transcripts in the intestine (Fig. 3; detailed data are shown in Additional file 2: Table S1) suggest that most genes involved in digestion or absorption, apoptosis, proliferation, regulation of gene expression, metabolism and other functions were down-regulated by fasting. The amounts of transcripts for these genes recovered hardly, partially or fully to the transcript amounts of the fed frogs within 1 day after refeeding. This suggests that the *Xenopus* intestine was functionally suppressed by fasting and that some genes respond quickly with refeeding for 1 day, although 1 day of refeeding is not enough for some genes to completely recover from the transcriptional down-regulation brought about by 3 weeks of fasting.

Out of 22 genes involved in digestive or absorptive function of the intestine, 19 genes were down-regulated by fasting. In the refed frogs, the expression levels of at least 7 genes (cd36, slc5a1.2, alpi1, alpi2, mgam, nts, and gip) remained low, and the expression levels of the others (fabp1, fabp2, fabp6, rbp2, slc2a5, slc15a1, slc16a3, anpep, vip, glp1, glp1r and vil1) recovered to variable extents. Such an expression pattern was also detected in other categories. In the category of “apoptosis”, caspase genes (casp1, casp3, casp7, casp8 and casp9) were down-regulated by fasting and their expression levels still remained low 1 day after refeeding. In the category of “proliferation”, the expression levels of all genes were down-regulated by fasting and partially recovered by refeeding. In the category of “regulation of gene expression”, all but four genes (thrb, ppard, rarb and rarg) were down-regulated by fasting and variably recovered by refeeding. All but the g6pc1 gene in the category of “metabolism” were down-regulated by fasting, and variably recovered by refeeding. The genes whose expression was quickly recovered nearly completely or up-regulated by 1-day refeeding were fabp1, fabp2, fabp6, rbp2, nos1, raptor, pcna, fxr, cdx2, ppargc1a, ppara, rara, pdk4, g6pc2, hmgcr, acadvl and lgr5. Conversely, the genes whose expression were not down-regulated by fasting and then recovered to the fed state levels or down-regulated by refeeding were fgf19, thrb, rarb, rarg and mex3a.

### Epigenetic changes of fasting- and refeeding-response genes in intestine

**Fig. 4:**
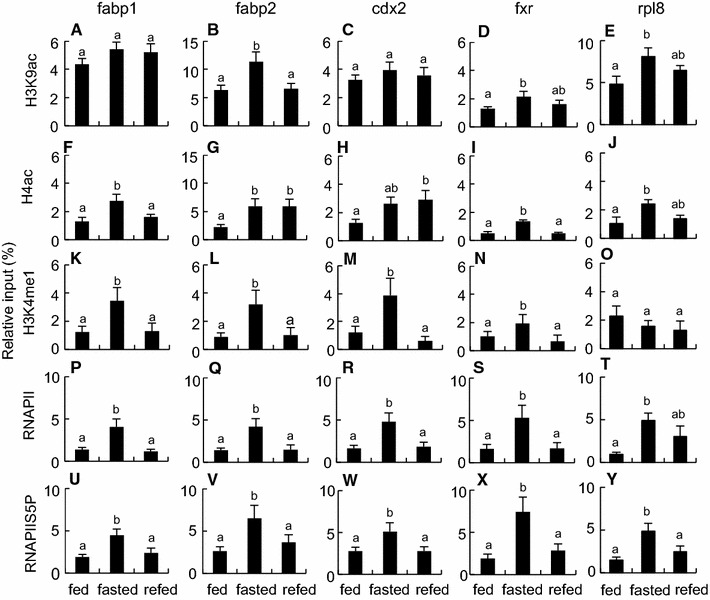
Epigenetic modifications on fabp1, fabp2, cdx2 and fxr genes in the intestines of fed, fasted and refed *X*. *laevi*s. Chromatin samples were prepared from the intestines of the frogs that were fed for 22 days (*fed*), fasted for 22 days (*fasted)*, or fasted for 21 days and then refed for 1 day (*refed*). Signals of ChIP on fabp1 (**A**, **F**, **K**, **P** and **U**), fabp2 (**B**, **G**, **L**, **Q** and **V**), cdx2 (**C**, **H**, **M**, **R** and **W**), fxr (**D**, **I**, **N**, **S** and **X**) and rpl8 (**E**, **J**, **O**, **T** and **Y**) genes were detected by qPCR following immunoprecipitation with antibodies against H3K9ac (**A**–**E**), H4ac (**F**–**J**), H3K4me1 (**K**–**O**), RNAPII (**P**–**T**) and RNAPIIS5P (**U**–**Y**). Primers used in qPCR are shown in Additional file 6: Table S3. Each value is the mean ± SEM (*n* = 8). *Distinct letters* denote significantly different means (*p* < 0.05). These experiments were repeated at least two times, with similar results

**Fig. 5:**
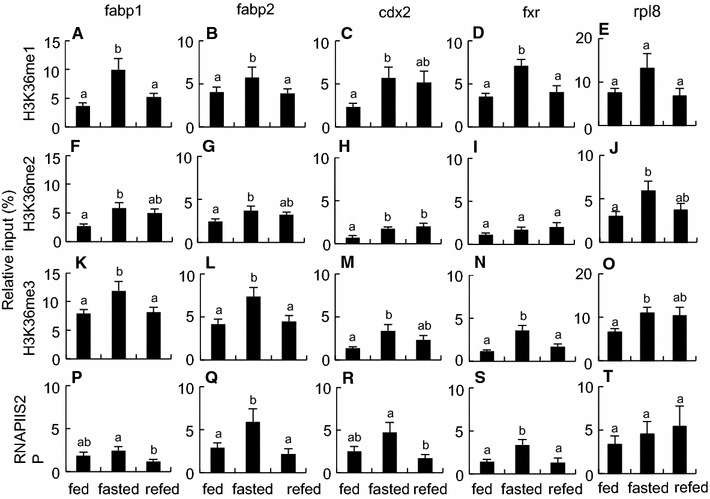
Epigenetic modifications on fabp1, fabp2, cdx2 and fxr genes in the intestines of fed, fasted and refed *X*. *laevi*s. Chromatin samples were prepared from the intestines from the frogs that were fed for 22 days (*fed*), fasted for 22 days (*fasted*), or fasted for 21 days and then refed for 1 day (*refed*). Signals of ChIP on fabp1 (**A**, **F**, **K** and **P** ), fabp2 (**B**, **G**, **L** and **Q**), cdx2 (**C**, **H**, **M** and **P**), fxr (**D**, **I**, **N** and **S**) and rpl8 (**E**, **J**, **O** and **T**) genes were detected by qPCR following immunoprecipitation with antibodies against H3K36me1 (**A**–**E**), H3K36me2 (**F**–**J**), H3K36me3 (**K**–**O**), and RNAPIIS2P (**P**–**T**). Primers used in qPCR are shown in Additional file 6: Table S3. Each value is the mean ± SEM (*n* = 8). *Distinct letters* denote significantly different means (*p* < 0.05). These experiments were repeated at least two times, with similar results

**Fig. 6:**
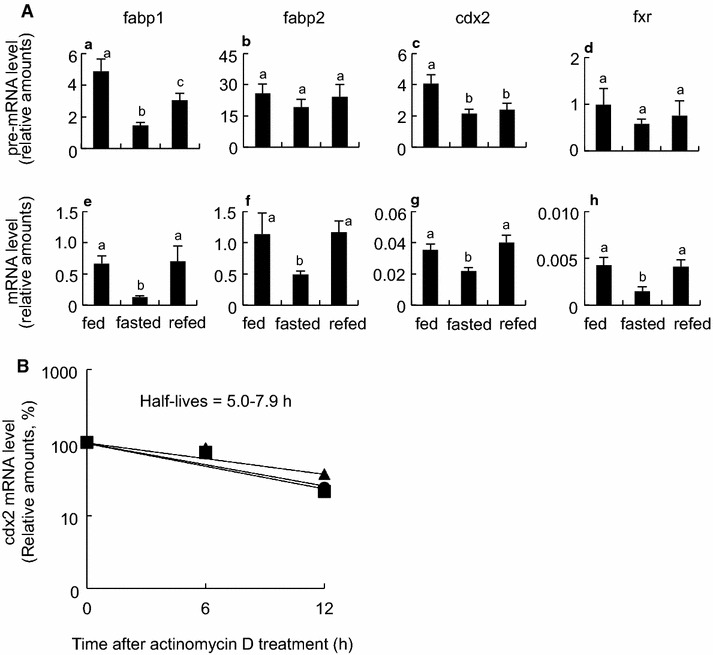
Post-transcriptional regulation of diet-response genes in the small intestine of *X*. *laevis.* **A** Comparison of pre-mRNA levels with matured mRNA levels of four fasting/refeeding-response genes. RNAs were prepared as shown in Fig. 3, and RT-qPCR (*n* = 8) was conducted using intron specific primers (*a*–*d*) and exon-specific primers (*e*–*h*) for fabp1 (*a* and *e*), fabp2 (*b* and *f*), cdx2 (*c* and *g)* and fxr (*d* and *h*) genes. Primers used in qPCR are shown in Additional file 6: Table S3. Each value is the mean ± SEM (*n* = 8). *Distinct letters* denote significantly different means (*p* < 0.05). **B** RT-qPCR of the cdx2 mRNA expression after treatment with actinomycin D. mRNAs were extracted from the primary tissue culture of the fed (*circle*), fasted (*triangle*) or refed (*square*) animals, as described in “Methods”. mRNA amounts at time 0 were set at 100 %. Depicted half lives were calculated using exponential regression. These experiments were repeated at least two times, with similar results

The fabp1, fabp2, cdx2 and fxr genes were selected for ChIP analysis as typical genes showing the down-regulation by fasting and the quick recovery by refeeding (Fig. 3). Our ChIP analysis revealed that these genes became epigenetically activated in the intestines with fasting and deactivated with refeeding (Figs. 4, 5), which had an inverse relationship with the transcript levels of these genes estimated by RT-qPCR (Fig. 6A).

Fasting for 22 days enhanced the amounts of acetylated histone H3 lysine 9 (H3K9ac) on the fabp2 and fxr genes, acetylated histone H4 (H4ac) on the fabp1, fabp2, and fxr genes, mono-methylated histone H3 lysine 4 (H3K4me1) on the four genes, and RNA polymerase II (RNAPII) and phosphorylated RNAPII serine 5 (RNAPIIS5P) on all four genes (Fig. 4), suggesting an activated state of the initiation/early elongation stages of transcription in these genes. Furthermore, fasting increased the amounts of mono-methylated histone H3 lysine 36 (H3K36me1) on all four genes, di-methylated histone H3 lysine 36 (H3K36me2) on the fabp1, fabp2 and cdx2 genes, tri-methylated histone H3 lysine 36 (H3K36me3) on all four genes, and phosphorylated RNAPII serine 2 (RNAPIIS2P) on the fabp2 and fxr genes (Fig. 5), revealing an activated state of the progressive elongation stage of transcription in these genes. Most of these epigenetic marks on the genes returned to the fed state within 1 day after refeeding. No effects of fasting and refeeding on the amounts of mono-, di-, and tri-methylated histone H3 lysine 9 (H3K9me1, H3K9me2 and H3K9me3, respectively) and di- and tri-methylated histone H3 lysine 4 (H3K4me2, and H3K4me3, respectively), were detected among the three groups (Additional file 3: Figure S2). The ChIP signals for pan-histones H3 and H4 did not differ among the three groups (Additional file 4: Figure S3). The ChIP signals for normal rabbit IgG were less than 1.1 % of input on all four genes and did not significantly differ among the three groups (Additional file 3: Figure S2).

Similar ChIP assays were conducted in the reference gene rpl8, whose expression levels were not different among the three groups. Fasting for 22 days enhanced the amounts of H3K9ac, H4ac, H3K36me2, H3K36me3, RNAPII and RNAPIIS5P, whereas refeeding for 1 day had only subtle effects on these modifications except for the amount of RNAPIIS2P (Figs. 4, 5). No significant differences were observed in the amounts of H3K4me1, H3K36me1, RNAPIIS2P and pan-histones H3 and H4 among the three groups (Figs. 4, 5; Additional file 4: Figure S3).

### Post-transcriptional regulation of fabp1, fabp2, cdx2 and fxr genes in intestine

Fasting decreased the amounts of the fabp1, fabp2, cdx2 and fxr pre-mRNAs to a lesser extent than those of the mature mRNAs (Fig. 6A). Refeeding recovered the amounts of the pre-mRNA to a lesser extent than those of the mature mRNAs to the fed state levels. Next, we investigated the decay of the cdx2 mRNA of the intestines that were cultured in the presence of actinomycin D after the frogs were fed, fasted, or re-fed. There were no significant differences in the mRNA decay among the three groups, with half-lives of 5.0–7.9 h (Fig. 6B).

## Discussion

The present study demonstrates that the *X. laevis* intestine responds to fasting and refeeding at epigenetic, transcriptional, and post-transcriptional levels, resulting in changes in the structure of the epithelial layer, intestinal specific functions, metabolism, and probably cell growth and differentiation. The remarkable features in the responses of the *X. laevis* intestine to fasting and refeeding are (1) an overall suppression and quick recovery of the transcription of genes associated with intestinal functions, as well as those of the epithelial layer structure, and (2) the discordance between the mRNA amounts (low when fasting and high when refeeding) and the states of epigenetic marks (an activated state when fasting and a basal state when refeeding) in the diet-response genes fabp1, fabp2, cdx2 and fxr. These features suggest that the *X*. *laevis* intestine has a mechanism by which, when fasting, the metabolic rate is suppressed at least through the transcriptional down-regulation of almost all of the genes to conserve energy, whereas, if once the frogs intake food, intestinal mass and functions can quickly recover to those of the fed state by epigenetic and/or post-transcriptional mechanisms, maintaining a standby mode during fasting periods.

### Structure of intestinal mucosa and gene expression

Our histological observations indicate that fasting and refeeding profoundly affect the structure of the mucosa where the epithelial cell differentiation, proliferation or apoptosis may be reduced with fasting and recovered with refeeding. In contrast to the muscularis externa, the mucosa is drastically changed by fasting and refeeding, in agreement with previous reports in fish [5], reptiles [11, 12] and other amphibians [13–15]. In particular, the epithelial layer that participates in intestinal specific functions, was down-regulated during fasting and recovered quickly by refeeding. Unlike the mucosa of the mammalian intestines, in which prolonged fasting causes severe atrophy [3] with impairment of mucosal barrier function [4], the structural integrity of the *X. laevis* intestine mucosa was maintained during fasting, even after 5 months of fasting. This notion was supported by changes in protein amounts of cell proliferation marker (PCNA) and in transcript amounts of the marker genes for stem cell (lgr5), epithelial cell differentiation (cdx2), cell proliferation (atm, pcna and components of mTOR signaling, mtor, raptor and rictor), whose expression levels correlated well with the morphological changes of the epithelial layer. The marker genes for apoptosis (casp1, casp3, casp7, casp8, casp9, and nos2) [16, 17] were also down-regulated by fasting but remained low at 1 day after refeeding. Under normal conditions, the epithelial cell turnover rate of the *X. laevis* intestine is approximately 16 days [18], 3–4 time longer than that in rats, which 4–5 days [1]. Fasting may further decelerate the usual cell turnover rate by decreasing both cell proliferation and apoptosis, although at present we cannot exclude the possibility that enterocyte hypertrophy contributes to the recovery from the intestinal atrophy as found in the intestines of the snake *Python molurus* [11] and the frog *Cyclorana alboguttata* [13]. A similar observation about the decline in both cell proliferation and apoptosis during fasting was also reported in the shark intestine [19]. Reduction in enterocyte number and in villus length with fasting [20], and enterocyte hypertrophy and hyperplasia with refeeding [21] may be common responses in the intestines of lower vertebrates.

A histologically interesting finding is that the intestine of the refed frogs had highly branched troughs in a villus-trough unit compared with the intestine of the fasted frogs, suggesting the appearance of the epithelial cells that begin to proliferate and differentiated at various positions in a villus-trough unit to generate new troughs within 1 day after refeeding. Future studies need to address what characteristics these cells have and how these cells appeared in response to refeeding.

### Intestinal functions and gene expression

Fasting down-regulated and refeeding recovered not only the functions of the intestinal epithelial cells but also the motility of the digestive tract. The activities of intestinal alkaline phosphatase, aminopeptidase, glucoamylase and maltase declined during fasting and quickly recovered to the feeding levels within 1 day after refeeding. Although the level of transcripts of these enzymes declined during fasting, the transcript levels of these enzymes hardly increased with refeeding, suggesting the presence of post-transcriptional regulation. Alternatively, as intestinal alkaline phosphatase, aminopeptidase and maltase-glucoamylase are membrane proteins that are subject to glycosylation, sulfation or phosphorylation at specific residues, regulation of such post-translational modifications may be another possibility to explain the discordance between the enzyme activity and transcription level. The expression of the fabp1, fabp2, fabp6 and rbp2 genes, which are involved in the cellular transport of fatty acids and retinol, was also quickly recovered or up-regulated from a down-regulated state of fasting within 1 day after refeeding. In contrast, mammalian intestines need at least 2–6 days after refeeding to recover the protein or transcript amounts of the alkaline phosphatase and glucose transporter, GLUT2 [22, 23]. The *X. laevis* intestine may have some mechanisms by which the enzyme activities and the transcription of the genes for fatty acid and retinol transport proteins respond quickly and selectively to refeeding. The presence of digesta in the rectum of the frogs that fasted for 5 months suggests an increase in digesta retention times during fasting. A similar observation has also been reported in the green striped burrowing frog, *Cyclorana alboguttata* during 3 months of estivation [24]. The low expression levels of gastrointestinal hormones such as nts, vip, glp1 and gip, which are activated in response to feeding, and nos1, which is involved in intestinal peristaltic movement [25], may contribute to the reduction in the motility of digestive tract during fasting.

### Metabolism and gene expression

During fasting, the intestine of *X. laevis* males may trigger the decline of the overall metabolic rate with monophasic transcriptional suppression to reduce the maintenance costs. During fasting, *X. laevis* females consume both glycogen stored in liver, ovaries and muscle, and lipids stored in fatty bodies and ovaries [26], with a reduction in oxygen consumption to about 30 % of the fed state. This phase lasts 4–6 months of fasting [8]. In a preliminary study using male *X. laevis* frogs, we could not detect the effects of 1 week-fasting on 19 of 21 transcript amounts and 3 of 4 biochemical parameters in plasma. The present study indicates that the plasma levels of glucose, triglycerides and free fatty acids declined or tended to decline and that most genes associated with metabolism that were tested are transcriptionally down-regulated with fasting for 22 days and 5 months. These changes in metabolites and transcripts we investigated during fasting periods correlate with a reduction in the metabolic rate [8]. Compared with two types of distinct metabolic responses to prolonged food deprivation, which are proposed on the basis of fuel availability in salamanders: successive phases (carbohydrate-, lipid-, and then lipid and protein-dominant catabolism) in a blind cave-dwelling salamander and monophase (linear and large decrease in all the energy reserves within the same time course) in a surface-dwelling salamander [27], the metabolic response of the *X. laevis* intestine seems to be monophasic at least during 5 months of fasting.

In rat fasted for 7 days, circulating glucose, triglyceride and cholesterol concentrations declined, whereas circulating free fatty acids increased, suggesting an increase in the mobilization of free fatty acids from tissues such as fatty bodies [28]. In the mouse intestine, fasting down- or up-regulates the gene expression of the following metabolic pathways with at least two successive phases [29]: amino acid metabolism (stimulation of glutamine synthesis by *Glul*), consumption of odd-chain fatty acids [activation of β-oxidation (*Acadvl* and *Hadha*) by *Ppara*], suppression of the glycolytic pathway (inhibition of pyruvate oxidation by *Pdk4*), activation of gluconeogenesis (*Pck1*) and the synthesis of ketone bodies on 12-h fasting; and the inhibition of glycolysis (*Ldh1*), stimulation of the glucose production from glutamate, and gluconeogenesis (*Pck1* and *G6pc*) on 3-day fasting. In contrast to the various patterns of gene expression in the mouse intestine, most of the genes tested in the *X. laevis* intestine were down-regulated after 22 days of fasting: in the glycolytic pathway (hk1, pk1r, pfkm), gluconeogenesis (pck1, pdk4, fbp1, g6pc1, and g6pc2), amino acid metabolism (glul), fatty acid oxidation (acox1, acox2, acadvl), and the pentose phosphate pathway (g6pd). Furthermore, unlike the mouse intestine, the *X. laevis* intestine did not show the shift in expression of the genes concerning energy metabolism. These observations suggest that there may be fundamental differences in the regulatory system of energy metabolism during fasting between the intestines from *X. laevis* and from mouse. It is likely that fasting for 22 days in *X. laevis* bring about metabolic rate depression, accompanied with a global reduction in many energy usages such as transcriptional and translational processes [9]. Interestingly, we detected several genes whose expression was not down-regulated, but rather up-regulated during fasting. These gene products act as transcription factors belonging to the nuclear receptor superfamily (Thrb, Ppard, Rarb, Rarg), signaling molecule (Fgf19) or RNA-binding protein delivering the target transcripts such as the cdx2 transcripts to P-body (Mex3a) [30], some of these gene products may be involved in the initiation of the metabolic rate depression on fasting in *X. laevis*.

### Epigenetic, transcriptional and post-transcriptional regulations

The most intriguing finding is that the transcript amounts of the fabp1, fabp2, cdx2 and fxr genes in the *X. laevis* intestine inversely correlated with euchromatin-associated epigenetic marks. This inverse relationship may not result from nucleosome removal that may occur in promoter regions of actively transcribed genes [31], because our fasting and refeeding conditions did not significantly affect the amounts of pan-histones H3 and H4 on the four genes. In the reference gene rpl8, the transcript amounts did not differ in the intestine among the fed, fasted and refed frogs. Nevertheless, most of euchromatin-associated epigenetic marks investigated were elevated on fasting. These findings raise the possibility that several euchromatin-associated epigenetic marks are elevated in many genes of the intestine on fasting regardless of whether the transcript amounts decrease or not. This suggests that fasting induces chromatin relaxation in the entire genome of the *X. laevis* intestine. Although similar global suppression of transcription was found in the liver and skeletal muscle of hibernating ground squirrels [32, 33], chromatin relaxation that is induced by increased histone acetylation did not occur. Nuclear run-on assays indicate decreased transcription initiation and elongation in the liver [32]. In the skeletal muscle, hibernation enhanced the amount of phosphorylated RNAPIIS5, suggesting the promoter-proximal pausing of RNAPII during hibernation [33]. A convincing body of evidence has accumulated showing that environmental signals such as heat shock and hypoxia modulate gene expression controlled by the promoter proximal pausing of RNAPII and its release [34, 35]. Suppression of transcription and epigenetic changes induced by these environmental signals imply that fasting induces RNAPII pausing in the *X. laevis* intestine and that refeeding acts as a signal to induce the release of paused RNAPII into active elongation, although there may be signal-dependent differences in epigenetic modifications.

In contrast, previous studies revealed good correlations between the increased transcription amounts and euchromatin-associated epigenetic marks in the rat genes (*Si*, *Rbp2*, *Slc2a5* and *Slc5a1*) involved in intestinal functions, under specific spatiotemporal or dietary conditions [36–38]. In nematode larvae, RNAPII accumulates on the promoters of growth and development genes with a decrease in mRNA amounts during fasting whereas transcriptional elongation promptly enhances with an increase in mRNA amounts in response to feeding [39]. In general, histone H4ac, H3K9ac, H3K4me1 and RNAPIIS5P are hallmarks from transcriptional initiation to early transcriptional elongation, whereas H3K36me3 and RNAPIIS2P are hallmarks of progressive stage of RNAPII transcriptional elongation [40]. The inverse or no relationships between transcript amounts and euchromatin-associated epigenetic marks, which we found in the *X. laevis* intestine, raise the possibility of the presence of transcriptional or post-transcriptional regulations at least in this amphibian species. Interestingly, the amounts of H3K4me3, one of euchromatin-associated epigenetic marks, and the amounts of H3K9me3 (Additional file 3: Figure S2), one of heterochromatin-associated epigenetic marks, hardly responded to fasting or refeeding on the fabp1, fabp2, cdx2 and fxr genes under our experimental conditions. It is likely that some of essential components for starting the transcription initiation or elongation (e.g. P-TEFb and BRD4) are lacking or inactive, or some inhibitory components (e.g. 7 SK-HEXIM inhibitory complex, NELF and DSIF) are recruited and still active into the complex of transcriptional machinery on fasting. We first considered the decay of the mature mRNA as a possible candidate for a regulatory step, we failed to detect significant differences in half-life of the cdx2 mature mRNA among the fed, fasted and refed frogs. The cdx2 pre-mRNA amounts, which were one or two order of magnitude less than the mature mRNA amounts, may be too small to estimate the half-lives by RT-qPCR. Alternatively, processing of pre-mRNA such as splicing, capping and polyadenylation, and the delivery system of the processed RNA species from nucleus to cytoplasm, are also possible regulatory steps [41]. Our observation that the amounts of the pre-mRNAs decreased by fasting and recovered by refeeding to lesser extents than did those of the mature mRNAs in all of the four genes tested is indicative of post-transcriptional regulation. At present it remains to be elucidated what molecular mechanism underlies by which transcript amounts are correlated inversely with euchromatin-associated epigenetic marks in the four selected genes. Even if the mRNA amounts of the genes that play an important role in the intestinal functions decreased during fasting, epigenetically active state on these genes could serve as a standby mode to respond quickly to unexpected food intake.

## Conclusions

This study demonstrates the down-regulation in the structure and functions of the *X. laevis* intestine by fasting and the quick recovery within 1 day after refeeding. However, several epigenetic marks on the diet-response genes suggest that the genes keep a transcriptionally active state under fasting conditions even if the transcription levels of the genes were suppressed. The euchromatin-associated epigenetic marks may positively contribute to the quick responses of the *X. laevis* intestine to refeeding at morphological, biochemical and transcriptional levels.

## Methods

### Reagents and antibodies

l-Alanine-4-nitroanilide (>99 % purity) was obtained from Sigma (St. Louis, MO). Actinomycin D (>95 % purity), kits for glucose, triglyceride, cholesterol and free fatty acid quantitation (Glucose CII Test Wako, Triglyceride E-test Wako Cholesterol E-test Wako and NEFA C-test Wako), 4-nitrophenol (>99 % purity), 4-nitroaniline (>99 % purity), maltose (>99 % purity), starch (enzyme assay grade) were purchased from Wako Pure Chemical Industries (Osaka, Japan). 4-Nitrophenyl phosphate was purchased from Kanto Chemical (Tokyo, Japan). Sulfadiazine (4-amino-*N*-pyrimidin- 2-yl-benzenesulfonamide, >98 % purity) was from LKT Laboratories (St. Paul, MN, USA).

Antibodies against C-terminal heptapeptide of wheat RNAPII (Cat. No. MMS-126R) and normal rabbit IgG (15006) were purchased from Covance (Berkeley, CA, USA), and Sigma, respectively. Antibodies against H3K4me1, H3K4me2 and H3K4me3 (ab8895, ab32356, and ab1012, respectively), H3K36me1, H3K36me2 and H3K36me3 (ab9048, ab9049 and ab9050, respectively), and RNAPIIS2P and RNAPIIS5P (ab5095 and ab5131, respectively) were obtained from Abcam (Tokyo, Japan). Antibodies against H3K9me1, H3K9me2 and H3K9me3 (07-450, 07-441, and 07-442, respectively), H4Ac that recognizes acetylated lysines 5, 8, 12 and 16 (06-598), H3K9ac (07-352), human pan-histone H3 (07-690) and pan-histoneH4 (05-857) were from Merck Millipore (Darmstadt, Germany). Antibodies against rat PCNA (M0879) and human α-tubulin (sc-5268) were obtained from Dako (Glostrup, Denmark) and Santa Cruz (Dallas, Texas, USA), respectively. The specificity of these antibodies is shown in Additional file 5: Table S2.

### Animal care, housing and experimental design

The African clawed frog *X. laevis* (male, 1 year old, 51–54 g) were obtained from Watanabe Breeding (Hyogo, Japan). Frogs were acclimated to laboratory conditions with feeding on 4 crickets (*Gryllus bimaculatus*, 16 mm in size and 0.3 g in weight) per frog every morning in aquaria (30 × 60 × 35 cm) filled with 15 L of dechlorinated tap water at 23 ± 1 °C under natural lighting conditions. After 1 week, 24 frogs were assigned to 1 of 3 groups (8 frogs/group) and the mean body mass of each group was adjusted to be similar at the beginning of the experiment. The frogs were (1) fed ad libitum 4 crickets/frog/day for 22 days (control), (2) fasted for 22 days, or (3) fasted for 21 days and then refed 4 crickets/frog for 1 day before sacrifice. During the experiment, the temperature was kept at 23 ± 1 °C under natural lighting conditions. In some experiments, frogs (n = 6) were fasted for 5 months. The rearing water was changed 3 times per week. The body mass of frogs was measured every week. At the end of the experiments, frogs were decapitated by guillotine, and blood was collected. Plasma was separated from blood cells by centrifugation at 3000*g* for 15 min at 4 °C and stored at −35 °C for later use. The gastrointestinal tract was removed and weighed, and its length was measured after the small intestine lumen was washed with ice-cold frog Ringer’s solution to remove digesta. The middle part of the intestine was used for morphological studies. Proximal and distal parts were mixed, and then used for RNA preparation, and ChIP and enzyme assays. For enzyme assays, after the lumen was rinsed with frog Ringer’s solution, small intestine was homogenized in 4 volumes of 10 mM potassium phosphate (K_2_HPO_4_/KH_2_PO_4_) buffer, pH 7.0, 1 mM phenylmethylsulfonyl fluoride and 1 mM benzamidine HCl in a Potter–Elvehjem homogenizer. Homogenate was stored at −84 °C until required.

All housing and experimental procedures were conducted in accordance with the code of ethics on the Animal Welfare Committee of Shizuoka University.

### Histology

Pieces (5 mm in length) of intestine were fixed in 4 % paraformaldehyde in phosphate buffered saline (PBS) overnight at room temperature. The fixed tissues were dehydrated through a graded ethanol series and embedded in Paraplast Plus (McCormick Scientific, St. Louis, MO, USA). Samples were transversely sectioned at 4 μm thickness using a microtome (Yamato Koki, Saitama, Japan). The specimens were stained with Mayer’s hematoxylin and eosin, dehydrated with ethanol, and then mounted in Entellan (Merck, Darmstadt, Germany), according to Nakakura et al. [42]. Stained sections were observed under a light microscope (BX61, Olympus, Tokyo, Japan). The intestinal diameter, the outer diameter of the mucosa/submucosa layer, the circumference of the epithelial layer, the width of the muscularis externa, the number of PAS-positive goblet cells (panel E in Additional file 1: Figure S1) and the number of troughs in a villus-trough unit were measured and using Image J (http://imagej.nih.gov/ij/), and calculated from three randomly selected sections per animal.

### Immunoblotting

Intestinal homogenates (60 μg) were run on 10 % sodium dodecyl sulfate–polyacrylamide gel electrophoresis. Proteins were transferred onto nitrocellulose membranes with a semidry transfer system for 2 h at 1.2 mA/cm^2^. The membranes were incubated in a 10 % skimmed milk in Tris-buffered saline (TBS), overnight at 4 °C and then probed with primary antibody against PCNA (1:1000) in 1 % skimmed milk in TBS for 1 h. After washing with TBS containing 0.1 % Tween 20 three times, blots were then incubated with horseradish peroxidase conjugated anti-mouse IgG (1:3000) for 0.5 h. Signals were developed by chemiluminescence (ECL reagent, GE Healthare, Piscataway, NJ). α-Tubulin level was used to normalize protein expression. Protein expression was quantified using a lumino-image analyzer (LAS-4000, Fuji Film, Tokyo, Japan).

### Measurements of plasma biochemical parameters

The plasma concentration of glucose was determined by the mutarotase-glucose oxidase method [43] using a kit (Glucose CII-testWako), triglycerides by the glycerol-3-phosphate oxidase method [44] using a kit (Triglyceride E-testWako), cholesterol by the cholesterol oxidase method [45] using a kit (Cholesterol E-testWako), and free fatty acids by the acyl-CoA synthetase and acyl-CoA oxidase method [46] using a kit (NEFA C-testWako) according to the manufacturer’s directions.

### Enzyme assays

Intestinal alkaline phosphatase activity was determined according to the Walter and Schutt method [47] with some modifications. Ten μL of the homogenate (diluted 1/40) was mixed with 90 μL of 6.7 mM 4-nitrophenyl phosphate in 1 M diethanolamine and 0.5 mM MgCl_2_, pH 9.8 (DM buffer). Mixtures were incubated for 1–5 min at 25 °C, and the increasing absorbance at 405 nm was monitored in a microplate reader (model Emax, Molecular Devices Corp., Sunnyvale, CA, USA). Based on a standard curve for 4-nitrophenol standards (0–0.08 mM in DM buffer), we calculated alkaline phosphatase activity in μmol 4-nitrophenol production/min/g protein.

Aminopeptidase assay was carried out using a synthetic substrate l-alanine-4-nitroanilide [48] with slight modifications. Ten μL of the homogenate (diluted 1/4) was mixed with 10 μL of 1.84 mM l-alanine-4-nitroanilide and 180 μL 60 mM phosphate buffer, pH 7.2, followed by incubating for 20 min at 25 °C. The liberated amount of 4-nitroaniline was determined from the increasing absorbance at 405 nm/min. Based on a standard curve for 4-nitroaniline standards (0–0.08 mM in phosphate buffer), activity was calculated as μmol 4-nitroaniline production/min/g protein.

Maltase activity was determined according to a method described previously [49] with some modifications. In brief, 10 μL of the homogenate (diluted 1/4 with 50 mM sodium phosphate, pH 6.5) was mixed with 10 μL of 56 mM maltose in 50 mM sodium phosphate, pH 6.5 for 60 min at 25 °C. The reaction was stopped by the addition of 30 μL of 0.5 M Tris–HCl, pH 7.0 and then boiled for 3 min. The solution was color-developed by the addition of 300 μL of the solution from the Glucose CII-testWako kit and incubation for 5 min at 37 °C on the basis of the glucose oxidase test [43]. Absorbance was measured at 490 nm with a microplate reader. Glucose standards (0–0.1 mg/mL) were also reacted with the solution of the kit. Based on a standard curve for glucose, we calculated maltase activity in μmol hydrolysed maltose/min/g protein.

Glucoamylase activity was determined by the method of Dahlqvist [50] with some modifications. Ten μL of the homogenate (diluted 1/4 with 50 mM sodium phosphate, pH 6.5) mixed with 10 μL of 2 % soluble starch in 50 mM sodium phosphate, pH 6.5 for 90 min at 25 °C. The reaction was stopped and liberated glucose was quantified as shown in the maltase assay.

The protein content of the intestinal homogenates was estimated by the micro-Lowry method [51] with bovine serum albumin (BSA) as the standard.

### RNA extraction and RT-qPCR analysis

Intestine (~0.1 g) was lysed with 1000 μL of the acid guanidinium thiocyanate solution [52]. Total RNA was isolated with phenol and chloroform. To confirm its integrity, RNA (1 μg per lane) was electrophoresed in a 1 % agarose gel containing 2.0 M formaldehyde, and 28S and 18S rRNAs were visualized by ethidium bromide staining. Reverse transcription was carried out using total RNA (200 ng) in 10 μL of 1 × Taqman RT buffer using Taqman RT reagents kit (Applied Biosystems, Foster City, CA, USA) according to the manufacturer’s instructions. The abundance of transcripts was estimated using Power SYBR Green Master Mix and ABI Prism 7000 sequence detection System (Applied Biosystems) with a specific primer set (each 200 nM) (Additional file 6: Table S3). Reactions were performed using the following protocol: 1 cycle of 50 °C (2 min) and 95 °C (10 min), and 40 cycles of 95 °C (15 s), 60 °C (1 min). Quantification was determined by applying the 2^−Cq^ formula and calculating the average of the values obtained for each sample. Eligibility of this formula was verified by qPCR using a RT-qPCR product of each transcript as a template at different concentrations that covered five orders of magnitude. To standardize each experiment, the abundance of the test transcripts was divided by that of ribosomal protein L8 (rpl8) in the same sample by the 2^−ΔΔCq^ method [53]. Detailed information about RT-qPCR is shown in Additional file 6: Tables S3 and Additional file 7: Table S4. The genes investigated here are functionally categorized into six groups: (1) digestion or absorption, (2) apoptosis, (3) proliferation, (4) regulation of gene expression, (5) metabolism and (6) others. To obtain clues about the post-transcriptional regulation of selected genes, the amounts of the pre-mRNAs and the mature mRNAs were quantified by RT-qPCR at the same time using the same intestinal samples.

To investigate the decay of specific mRNAs under conditions where *de novo* RNA synthesis is inhibited, the intestine was briefly washed in PBS containing 0.5 mg/mL sulfadiazine, longitudinally cut into four pieces (approximately 3 × 0.2 cm), and then cultured in 70 % Leibovitz’s L-15 medium containing 10 % fetal bovine serum in the presence or absence of 25 μg/mL actinomycin D for 0, 6, 12 and 24 h at 25 °C with air. Total RNA was extracted from the piece of the tissue cultured, followed by RT-qPCR, as described above.

### ChIP assay

ChIP assay was performed as previously described [54]. In brief, chromatin in tissues was cross-linked in 10 mL of fixation solution (1 % formaldehyde, 4.5 mM HEPES, pH 8.0, 9 mM NaCl, 0.09 mM EDTA, and 0.04 mM EGTA) at 25 °C. After 15 min, 1.5 M glycine was added up to one-tenth the volume of the fixation solution to stop the cross-linking. Tissues were washed twice with 10 mL of FACS solution (1 × PBS, 2 % bovine serum and 0.05 % sodium azide). The pelleted sample was solubilized in 1 mL of lysis buffer (50 mM Tris–HCl, pH 8.0, 10 mM EDTA, 1 % sodium dodecyl sulfate). The lysate was sonicated (12 × 30-s pulses; Ultra5 homogenizer, VP-55, TAITEC, Saitama, Japan) to obtain DNA fragments. The diluted chromatin sample (1 mL) was mixed with 50 % Protein G Sepharose slurry (10 μL) containing 100 μg/ml salmon sperm DNA and 1 % BSA. The supernatant was used for input (50 μL) and each ChIP assay (225 μL). Chromatin solution was incubated in a rotator at 4 °C overnight with each antibody (0.3–0.6 μg depending antibodies used) or normal IgG (3.0 μg). The 50 % Protein G Sepharose/salmon sperm DNA/BSA slurry (5 μL) was added to the chromatin-antibody solution. The immunoreactive chromatin was recovered in 400 μL of ChIP direct elution buffer (10 mM Tris–HCl, pH 8.0, 300 mM NaCl, 5 mM EDTA and 0.5 % sodium dodecyl sulfate). After reverse cross-linking by heating the samples at 65 °C overnight and treating with 50 μg/mL Proteinase K and 10 μg/mL ribonuclease A, one twenty-fifth of the extracted DNA was subjected to qPCR using primer sets (Additional file 6: Table S3) to quantify the amounts of DNA. The C_q_ values of the ChIP signals were expressed as percentages of the ChIP signals for the input DNA. Detailed information about qPCR is shown in Additional file 6: Tables S3 and Additional file 7: Table S4.

### Statistical analysis

All assay data are presented as mean ± standard error of the mean (SEM). Differences between groups were analyzed by one-way analysis of variance using the Fisher’s least significant difference test for multiple comparisons to show significant differences. *p* < 0.05 was considered statistically significant.

## Additional files

10.1186/s13578-016-0067-9 Histochemical localization of alkaline phosphatase activity and PAS-positive goblet cells in the intestine of fed, fast or refed *X. laevis.* The intestines were collected from frogs that were fed for 22 days (*A and E*), fasted for 22 days (*B*), fasted for 21 days and then refed for 1 day (*C*) and fasted for 5 months (*D*). *A*-*D*; alkaline phosphatase activity; *E*; PAS-staining. el; epitherial layer; lu, lumen; gc; goblet cells. Bar, 500 μm (*A*-*D*) and 50 μm (*E*).
10.1186/s13578-016-0067-9 The list of the relative gene expressions by RT-qPCR.
10.1186/s13578-016-0067-9 Epigenetic modifications on fabp1, fabp2, cdx2 and fxr genes in the intestines of fed, fasted and refed *X.* *laevis*. Chromatin samples were prepared from the intestines from animals that were fed for 22 days (*fed*), fasted for 22 days (*fasted*), and fasted for 21 days and then refed for 1 day (*refed*). Signals of ChIP on fabp1 (*A, E, I, M, Q* and *U*), fabp2 (*B, F, J, N, R* and *V*), cdx2 (*C, G K, O, S* and *W*), fxr (*D, H, L, P, T* and *X*) genes were detected by qPCR following immunoprecipitation with antibodies against H3K9me1 (*A-D*), H3K9me2 (*E-H*), H3K9me3 (*I-L*), H3K4me2 (*M-P*), H3K4me3 (*Q*-*T*), and normal IgG (*U-X*). Primers used in qPCR are shown in Additional file 5: Table S2. Each value is the mean ± SEM (*n* = 8). Distinct letters denote significantly different means, and were determined by one-way analysis of variance and Fisher’s least significant difference test for multiple comparisons (*p* < 0.05).
10.1186/s13578-016-0067-9 Amounts of pan-histones H3 and H4 on fabp1, fabp2, cdx2, fxr and rpl8 genes in the intestines of fed, fasted and refed *X. laevis*. Chromatin samples were prepared from the intestines from animals that were fed for 22 days (*fed*), fasted for 22 days (*fasted*), and fasted for 21 days and then refed for 1 day (*refed*). Signals of ChIP on fabp1 (*A* and *F*), fabp2 (*B* and *G*), cdx2 (*C* and *H*), fxr (*D* and *I*) and rpl8 (*E* and *J*) genes were detected by qPCR following immunoprecipitation with antibodies against pan-H3 (*A-E*) and pan-H4 (*F-J*). Primers used in qPCR are shown in Additional file 5: Table S2. Each value is the mean ± SEM (*n* = 8). Distinct letters denote significantly different means, and were determined by one-way analysis of variance and Fisher’s least significant difference test for multiple comparisons (*p* < 0.05).
10.1186/s13578-016-0067-9 Specificity of antibodies used in this study.
10.1186/s13578-016-0067-9 The list of primers used and efficiencies in quantitative polymerase chain reaction.
10.1186/s13578-016-0067-9 Detailed qPCR information.

## Abbreviations

BSA: bovine serum albumin
ChIP: chromatin immunoprecipitation
H3K4me1: monomethylated histone H3 lysine 4
H3K4me2: dimethylated histone H3 lysine 4
H3K4me3: trimethylated histone H3 lysine 4
H3K9me1: monomethylated histone H3 lysine 9
H3K9me2: dimethylated histone H3 lysine 9
H3K9me3: trimethylated histone H3 lysine 9
H3K36me1: monomethylated histone H3 lysine 36
H3K36me2: dimethylated histone H3 lysine 36
H3K36me3: trimethylated histone H3 lysine 36
H4Ac: acetylated histone H4
H3K9ac: acetylated histone H3 lysine 9
PCNA: proliferating cell nuclear antigen
PBS: phosphate buffered saline
RNAPII: RNA polymerase II
RNAPIIS2P: phosphorylated RNAPII serine 2
RNAPIIS5P: phosphorylated RNAPII serine 5
RT-qPCR: reverse transcription-quantitative polymerase chain reaction
SEM: standard error of the mean
TBS: Tris-buffered saline
