# Supplementary material for: Morphological, biochemical, transcriptional and epigenetic responses to fasting and refeeding in intestine of Xenopus laevis
Source: Cell Biosci. 2016 Jan 21;6:2. doi: 10.1186/s13578-016-0067-9 (PMC4721045; doi:10.1186/s13578-016-0067-9)
Supplement: Supplementary file 1 — 10.1186/s13578-016-0067-9 Histochemical localization of alkaline phosphatase activity and PAS-positive goblet cells in the intestine of fed, fast or refed X. laevis. The intestines were collected from frogs that were fed for 22 days (A and E), fasted for 22 days (B), fasted for 21 days and then refed for 1 day (C) and fasted for 5 months (D). A-D; alkaline phosphatase activity; E; PAS-staining. el; epitherial layer; lu, lumen; gc; goblet cells. Bar, 500 μm (A-D) and 50 μm (E). [file 13578_2016_67_MOESM1_ESM.pdf]

Figure S1

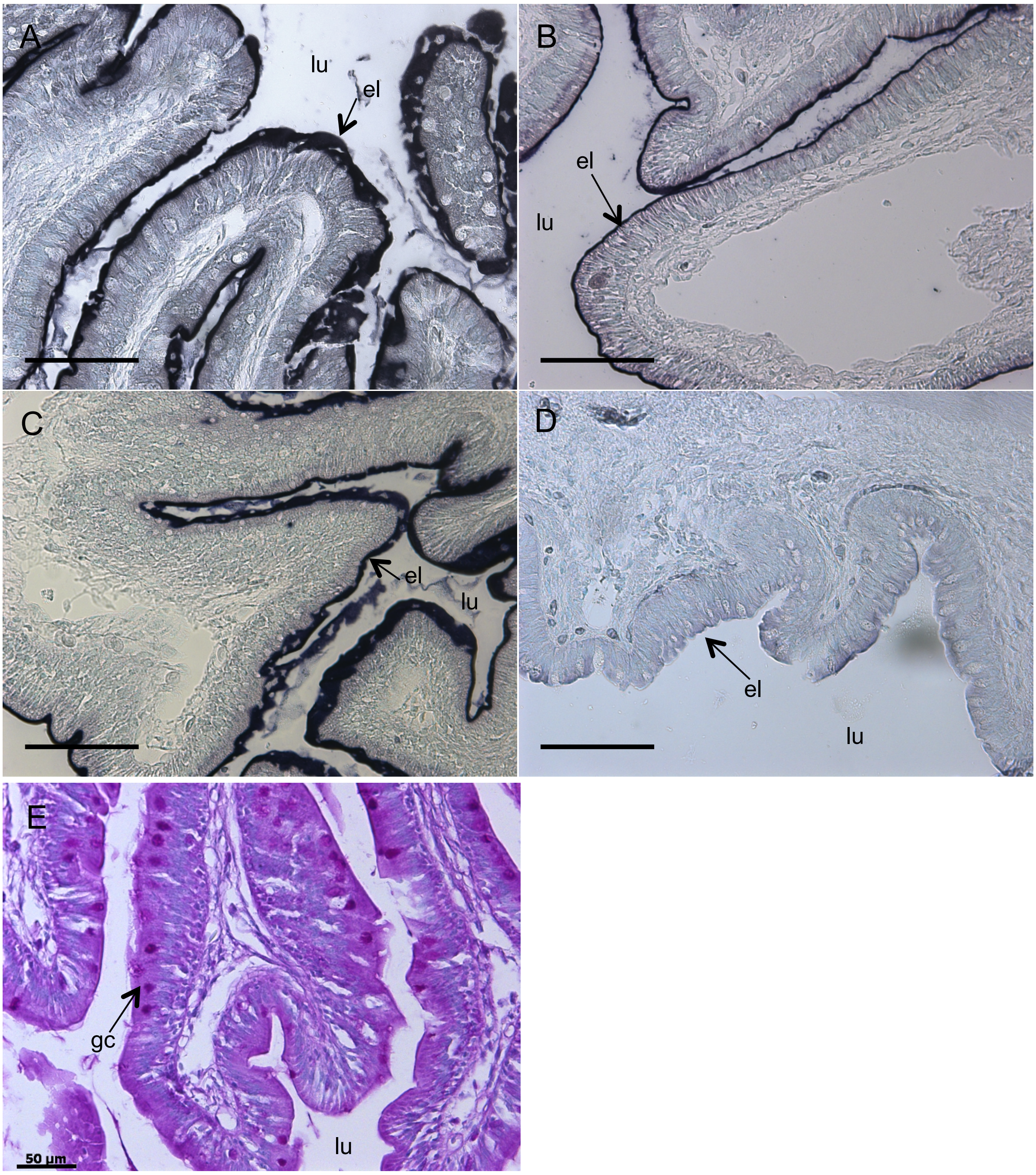

Fig. S1. Histochemical localization of alkaline phosphatase activity and PAS-positive goblet cells in the intestine of fed, fast or refed *X. laevis.* The intestines were collected from frogs that were fed for 22 days (*A and E*), fasted for 22 days (*B*), fasted for 21 days and then refed for 1 day (*C*) and fasted for 5 months (*D*). *A-D*; alkaline phosphatase activity; *E*; PAS-staining. el; epitherial layer; lu, lumen; gc; goblet cells. Bar, 500 µm (*A-D*) and 50 µm (*E*).

Alkaline phosphatase histochemistry was carried out according to the Cox and Singer method (1999). Fixed tissue sections were soaked in 0.2% Tween-20/PBS for 10 min and then rinsed in PBS for 10 min. Sections were incubated with the labeling reaction solution contained 0.17 mg/ml 5-bromo-4-chloro-3-indolyl-phosphate and 0.33 mg/ml nitro blue tetrazolium in reaction buffer (100 mM Tris-HCl, 100 mM NaCl, 5 mM $MgCl_2$, pH 9.5) for 30 min in the dark. De-waxed sections were stained with periodic acid-Schiff-hematoxylin (PAS-H) to localize the goblet cells (McManus, 1948).

Cox WG, Singer VL. A High-resolution, fluorescence-based method for localization of endogenous alkaline phosphatase activity. J Histochem Cytochem. 1999;47(11):1443–55.
McManus JF. Histological and histochemical uses of periodic acid. Stain Technol. 1948;23(3):99-108.
