# Supplementary material for: Morphological, biochemical, transcriptional and epigenetic responses to fasting and refeeding in intestine of Xenopus laevis
Source: Cell Biosci. 2016 Jan 21;6:2. doi: 10.1186/s13578-016-0067-9 (PMC4721045; doi:10.1186/s13578-016-0067-9)
Supplement: Supplementary file 2 — 10.1186/s13578-016-0067-9 The list of the relative gene expressions by RT-qPCR. [file 13578_2016_67_MOESM2_ESM.docx]

| Table S1. The list of the relative gene expressions by RT-qPCR. | | | | | | | | | |  |  |  |  |  |  |  |
| --- | --- | --- | --- | --- | --- | --- | --- | --- | --- | --- | --- | --- | --- | --- | --- | --- |
|  | Each mRNA levels/rpl8 mRNA ± SEM | | | | | | | | | | |  |  | *p* value | | |
| gene | fed | | |  | fasted | | |  | refed | | |  |  | fed v.s. fasted | fed v.s. refed | fasted v.s. refed |
| fabp1 | 0.4801 | ± | 0.0484 | a | 0.1303 | ± | 0.0188 | b | 0.4645 | ± | 0.0818 | a |  | 0.0001 | 0.8379 | 0.0002 |
| fabp2 | 1.1405 | ± | 0.3321 | a | 0.4899 | ± | 0.0559 | b | 1.1684 | ± | 0.1778 | a |  | 0.0486 | 0.9315 | 0.0471 |
| fabp6 | 4.4576 | ± | 1.4896 | a | 0.8008 | ± | 0.1933 | b | 5.6729 | ± | 0.8009 | a |  | 0.0219 | 0.4029 | 0.0035 |
| rbp2 | 4.3304 | ± | 0.7973 | a | 1.0428 | ± | 0.3225 | b | 10.5358 | ± | 0.5210 | c |  | 0.0011 | 0.0001> | 0.0001> |
| cd36 | 0.4396 | ± | 0.0518 | a | 0.0479 | ± | 0.0128 | b | 0.0289 | ± | 0.0048 | b |  | 0.0001> | 0.0001> | 0.6477 |
| slc2a5 | 0.3105 | ± | 0.0417 | a | 0.1823 | ± | 0.0395 | b | 0.1600 | ± | 0.0124 | b |  | 0.0159 | 0.0033 | 0.6511 |
| slc5a1.2 | 0.0115 | ± | 0.0013 | a | 0.0029 | ± | 0.0007 | b | 0.0040 | ± | 0.0006 | b |  | 0.0001> | 0.0001> | 0.4206 |
| slc10a2 | 0.0006 | ± | 0.0001 | a | 0.0005 | ± | 0.0002 | a | 0.0006 | ± | 0.0001 | a |  | 0.7778 | 0.9973 | 0.7655 |
| slc15a1 | 12.3450 | ± | 1.6679 | a | 4.8499 | ± | 1.7095 | b | 5.8726 | ± | 0.5529 | b |  | 0.0014 | 0.0024 | 0.6150 |
| slc16a3 | 0.0107 | ± | 0.0013 | a | 0.0045 | ± | 0.0018 | b | 0.0057 | ± | 0.0009 | b |  | 0.0043 | 0.0135 | 0.5560 |
| alpi1 | 0.9127 | ± | 0.1101 | a | 0.3367 | ± | 0.0542 | b | 0.2353 | ± | 0.0278 | b |  | 0.0001> | 0.0001> | 0.3520 |
| alpi2 | 0.0191 | ± | 0.0045 | a | 0.0045 | ± | 0.0010 | b | 0.0064 | ± | 0.0015 | b |  | 0.0071 | 0.0074 | 0.7008 |
| anpep | 5.0864 | ± | 0.9261 | a | 0.3774 | ± | 0.1437 | b | 3.0032 | ± | 0.4748 | c |  | 0.0001 | 0.0316 | 0.0140 |
| mgam | 4.2256 | ± | 0.7880 | a | 1.3282 | ± | 0.3782 | b | 1.1212 | ± | 0.0634 | b |  | 0.0009 | 0.0003 | 0.7828 |
| nts | 0.7407 | ± | 0.0659 | a | 0.1149 | ± | 0.0348 | b | 0.2560 | ± | 0.0331 | b |  | 0.0001> | 0.0001> | 0.0658 |
| vip | 0.0329 | ± | 0.0015 | a | 0.0059 | ± | 0.0010 | b | 0.0199 | ± | 0.0017 | c |  | 0.0001> | 0.0001> | 0.0001> |
| sst | 0.1341 | ± | 0.0167 | a | 0.1213 | ± | 0.0266 | a | 0.1708 | ± | 0.0269 | a |  | 0.7100 | 0.2751 | 0.1595 |
| glp1 | 0.0326 | ± | 0.0055 | a | 0.0072 | ± | 0.0024 | b | 0.0226 | ± | 0.0027 | a |  | 0.0002 | 0.0791 | 0.0125 |
| glp1r | 0.0534 | ± | 0.0073 | a | 0.0177 | ± | 0.0029 | b | 0.0289 | ± | 0.0028 | b |  | 0.0001 | 0.0023 | 0.1510 |
| gip | 0.1663 | ± | 0.0162 | a | 0.0368 | ± | 0.0069 | b | 0.0570 | ± | 0.0036 | b |  | 0.0001> | 0.0001> | 0.2508 |
| fgf19 | 0.0317 | ± | 0.0090 | a | 0.0474 | ± | 0.0179 | a | 0.0314 | ± | 0.0090 | a |  | 0.6559 | 0.9998 | 0.6451 |
| vil1 | 3.2816 | ± | 0.4378 | a | 0.5185 | ± | 0.2110 | b | 1.9979 | ± | 0.2480 | c |  | 0.0001> | 0.0095 | 0.0046 |
|  |  |  |  |  |  |  |  |  |  |  |  |  |  |  |  |  |
| casp1 | 0.0452 | ± | 0.0078 | a | 0.0063 | ± | 0.0019 | b | 0.0102 | ± | 0.0014 | b |  | 0.0001> | 0.0001> | 0.5430 |
| casp3 | 0.1555 | ± | 0.0252 | a | 0.0359 | ± | 0.0134 | b | 0.0354 | ± | 0.0037 | b |  | 0.0001 | 0.0001 | 0.9813 |
| casp7 | 0.0250 | ± | 0.0045 | a | 0.0110 | ± | 0.0033 | b | 0.0106 | ± | 0.0018 | b |  | 0.0095 | 0.0064 | 0.9395 |
| casp8 | 0.0137 | ± | 0.0029 | a | 0.0030 | ± | 0.0013 | b | 0.0067 | ± | 0.0009 | b |  | 0.0008 | 0.0143 | 0.1706 |
| casp9 | 0.0327 | ± | 0.0048 | a | 0.0138 | ± | 0.0071 | b | 0.0126 | ± | 0.0017 | b |  | 0.0126 | 0.0067 | 0.8597 |
| nos1 | 0.0106 | ± | 0.0015 | a | 0.0077 | ± | 0.0020 | a | 0.0113 | ± | 0.0005 | a |  | 0.1772 | 0.6963 | 0.1014 |
| nos2 | 0.0049 | ± | 0.0008 | a | 0.0035 | ± | 0.0012 | ab | 0.0022 | ± | 0.0004 | b |  | 0.2599 | 0.0255 | 0.2514 |
|  |  |  |  |  |  |  |  |  |  |  |  |  |  |  |  |  |
| mtor | 0.0398 | ± | 0.0050 | a | 0.0106 | ± | 0.0032 | b | 0.0337 | ± | 0.0057 | a |  | 0.0005 | 0.3782 | 0.0035 |
| mlst8 | 0.0208 | ± | 0.0032 | a | 0.0126 | ± | 0.0030 | b | 0.0138 | ± | 0.0014 | ab |  | 0.0420 | 0.0597 | 0.7455 |
| raptor | 0.0267 | ± | 0.0052 | a | 0.0120 | ± | 0.0040 | b | 0.0324 | ± | 0.0027 | a |  | 0.0334 | 0.3139 | 0.0048 |
| rictor | 0.0734 | ± | 0.0093 | a | 0.0171 | ± | 0.0023 | b | 0.0546 | ± | 0.0034 | c |  | 0.0001> | 0.0414 | 0.0007 |
| mapkap1 | 0.0995 | ± | 0.0179 | a | 0.0385 | ± | 0.0120 | b | 0.0514 | ± | 0.0062 | b |  | 0.0077 | 0.0149 | 0.5324 |
| atm | 0.1157 | ± | 0.0091 | a | 0.0311 | ± | 0.0115 | b | 0.0817 | ± | 0.0087 | a |  | 0.0001> | 0.0197 | 0.0016 |
| mki67 | 0.1192 | ± | 0.0136 | a | 0.0132 | ± | 0.0032 | b | 0.0534 | ± | 0.0069 | c |  | 0.0001> | 0.0001> | 0.0073 |
| pcna | 0.0809 | ± | 0.0127 | a | 0.0188 | ± | 0.0081 | b | 0.0726 | ± | 0.0106 | a |  | 0.0007 | 0.5852 | 0.0024 |
|  |  |  |  |  |  |  |  |  |  |  |  |  |  |  |  |  |
| fxr | 0.0043 | ± | 0.0009 | a | 0.0015 | ± | 0.0005 | b | 0.0041 | ± | 0.0007 | a |  | 0.0122 | 0.8897 | 0.0166 |
| cdx1 | 2.4648 | ± | 0.2375 | a | 0.5318 | ± | 0.0639 | b | 1.2751 | ± | 0.1209 | c |  | 0.0001> | 0.0001 | 0.0080 |
| cdx2 | 0.0353 | ± | 0.0040 | a | 0.0218 | ± | 0.0021 | b | 0.0402 | ± | 0.0047 | a |  | 0.0193 | 0.3603 | 0.0023 |
| creb1 | 0.0769 | ± | 0.0122 | a | 0.0219 | ± | 0.0050 | b | 0.0508 | ± | 0.0078 | a |  | 0.0004 | 0.0517 | 0.0390 |
| lxra | 0.2796 | ± | 0.0376 | a | 0.0615 | ± | 0.0089 | b | 0.1594 | ± | 0.0113 | c |  | 0.0001> | 0.0030 | 0.0194 |
| thrb | 0.0269 | ± | 0.0029 | a | 0.0603 | ± | 0.0252 | a | 0.0223 | ± | 0.0021 | a |  | 0.1158 | 0.8151 | 0.0675 |
| ppargc1a | 0.0459 | ± | 0.0043 | a | 0.0334 | ± | 0.0102 | a | 0.0470 | ± | 0.0040 | a |  | 0.2091 | 0.9107 | 0.1640 |
| nr3c1 | 0.2923 | ± | 0.0461 | a | 0.1020 | ± | 0.0231 | b | 0.1421 | ± | 0.0134 | b |  | 0.0004 | 0.0025 | 0.3847 |
| hnf1a | 0.6268 | ± | 0.1355 | a | 0.1893 | ± | 0.0315 | b | 0.4992 | ± | 0.0864 | ab |  | 0.0091 | 0.3714 | 0.0536 |
| hnf1b | 0.0005 | ± | 0.0001 | a | 0.0002 | ± | 0.0001 | b | 0.0002 | ± | 0.0000 | b |  | 0.0014 | 0.0003 | 0.5997 |
| hnf4a | 0.3055 | ± | 0.0556 | a | 0.0584 | ± | 0.0123 | b | 0.2398 | ± | 0.0335 | a |  | 0.0011 | 0.2531 | 0.0087 |
| ppara | 0.0505 | ± | 0.0080 | a | 0.0169 | ± | 0.0043 | b | 0.0656 | ± | 0.0072 | a |  | 0.0028 | 0.1256 | 0.0001 |
| ppard | 0.0384 | ± | 0.0044 | a | 0.0403 | ± | 0.0100 | a | 0.0154 | ± | 0.0015 | b |  | 0.8168 | 0.0078 | 0.0061 |
| rara | 0.3310 | ± | 0.0285 | a | 0.1561 | ± | 0.0287 | b | 0.3524 | ± | 0.0314 | a |  | 0.0009 | 0.6098 | 0.0002 |
| rarb | 0.0166 | ± | 0.0029 | a | 0.0675 | ± | 0.0335 | a | 0.0166 | ± | 0.0012 | a |  | 0.0720 | 0.9990 | 0.0637 |
| rarg | 0.0463 | ± | 0.0060 | a | 0.1305 | ± | 0.0593 | a | 0.0380 | ± | 0.0042 | a |  | 0.0756 | 0.8496 | 0.0527 |
| rxra | 0.0660 | ± | 0.0098 | a | 0.0230 | ± | 0.0048 | b | 0.0403 | ± | 0.0061 | b |  | 0.0006 | 0.0211 | 0.1197 |
| rxrb | 0.0307 | ± | 0.0037 | a | 0.0079 | ± | 0.0015 | b | 0.0207 | ± | 0.0027 | c |  | 0.0001> | 0.0215 | 0.0056 |
| rxrg | 0.0062 | ± | 0.0009 | a | 0.0019 | ± | 0.0002 | b | 0.0028 | ± | 0.0006 | b |  | 0.0005 | 0.0018 | 0.3984 |
|  |  |  |  |  |  |  |  |  |  |  |  |  |  |  |  |  |
| hk1 | 0.0258 | ± | 0.0027 | a | 0.0069 | ± | 0.0017 | b | 0.0226 | ± | 0.0019 | a |  | 0.0001> | 0.3085 | 0.0001> |
| pklr | 0.5312 | ± | 0.0572 | a | 0.1393 | ± | 0.0160 | b | 0.2514 | ± | 0.0226 | b |  | 0.0001> | 0.0001 | 0.0686 |
| pkfma | 0.1333 | ± | 0.0164 | a | 0.0190 | ± | 0.0024 | b | 0.0496 | ± | 0.0031 | b |  | 0.0001> | 0.0001> | 0.0647 |
| pdk4 | 0.1293 | ± | 0.0198 | a | 0.0463 | ± | 0.0110 | b | 0.2100 | ± | 0.0302 | c |  | 0.0257 | 0.0198 | 0.0001 |
| pck1 | 0.0167 | ± | 0.0039 | a | 0.0064 | ± | 0.0022 | b | 0.0110 | ± | 0.0019 | ab |  | 0.0149 | 0.1432 | 0.2116 |
| fbp1 | 1.1829 | ± | 0.1587 | a | 0.2284 | ± | 0.0236 | b | 0.3037 | ± | 0.0390 | b |  | 0.0001> | 0.0001> | 0.6266 |
| g6pc | 0.0044 | ± | 0.0009 | a | 0.0048 | ± | 0.0012 | a | 0.0051 | ± | 0.0011 | a |  | 0.0755 | 0.9715 | 0.0808 |
| g6pc2 | 0.1771 | ± | 0.0281 | a | 0.1306 | ± | 0.0293 | a | 0.2757 | ± | 0.0380 | b |  | 0.3112 | 0.0395 | 0.0053 |
| glul | 2.3741 | ± | 0.3525 | a | 0.8084 | ± | 0.1163 | b | 1.0929 | ± | 0.1561 | b |  | 0.0003 | 0.0009 | 0.4089 |
| hmgcr | 0.2944 | ± | 0.0758 | a | 0.0909 | ± | 0.0161 | b | 0.2901 | ± | 0.0300 | a |  | 0.0153 | 0.9523 | 0.0173 |
| apoa1 | 42.7757 | ± | 5.4732 | a | 2.2747 | ± | 0.6472 | b | 10.2983 | ± | 1.4550 | b |  | 0.0001> | 0.0001> | 0.1192 |
| hadha | 0.3204 | ± | 0.0321 | a | 0.0490 | ± | 0.0078 | b | 0.1941 | ± | 0.0239 | c |  | 0.0001> | 0.0017 | 0.0010 |
| acox1 | 1.8204 | ± | 0.2841 | a | 0.3975 | ± | 0.0462 | b | 1.3817 | ± | 0.1027 | a |  | 0.0001 | 0.1091 | 0.0024 |
| acox2 | 0.2525 | ± | 0.0543 | a | 0.1055 | ± | 0.0310 | b | 0.1606 | ± | 0.0154 | ab |  | 0.0152 | 0.1116 | 0.3455 |
| acadvl | 0.0432 | ± | 0.0097 | a | 0.0165 | ± | 0.0047 | b | 0.0657 | ± | 0.0066 | a |  | 0.0282 | 0.0503 | 0.0005 |
| g6pd | 0.3530 | ± | 0.0416 | a | 0.1002 | ± | 0.0296 | b | 0.1061 | ± | 0.0113 | b |  | 0.001> | 0.0001> | 0.8931 |
|  |  |  |  |  |  |  |  |  |  |  |  |  |  |  |  |  |
| mex3a | 0.0082 | ± | 0.0003 | a | 0.0107 | ± | 0.0051 | a | 0.0051 | ± | 0.0004 | a |  | 0.5139 | 0.3712 | 0.1350 |
| lgr5 | 0.0133 | ± | 0.0020 | a | 0.0026 | ± | 0.0006 | b | 0.0152 | ± | 0.0015 | a |  | 0.0002 | 0.3914 | 0.0001> |
| RNA samples were prepared from the intestines of animals that were fed for 22 days (fed), fasted for 22 days (fasted), and fasted for 21 days and then refed for 1 day (refed). Primers used in qPCR are shown in Table S2. Each genes expression was standardized by rpl8 gene expression. Values presented are means ± SEM (n = 8). Distinct letters denote significantly different means, and were determined by one-way analysis of variance and Fisher’s least significant difference test for multiple comparisons (*p* < 0.05). | | | | | | | | | | | | | | | | |
