# Supplementary material for: Morphological, biochemical, transcriptional and epigenetic responses to fasting and refeeding in intestine of Xenopus laevis
Source: Cell Biosci. 2016 Jan 21;6:2. doi: 10.1186/s13578-016-0067-9 (PMC4721045; doi:10.1186/s13578-016-0067-9)
Supplement: Supplementary file 3 — 10.1186/s13578-016-0067-9 Epigenetic modifications on fabp1, fabp2, cdx2 and fxr genes in the intestines of fed, fasted and refed X. laevis. Chromatin samples were prepared from the intestines from animals that were fed for 22 days (fed), fasted for 22 days (fasted), and fasted for 21 days and then refed for 1 day (refed). Signals of ChIP on fabp1 (A, E, I, M, Q and U), fabp2 (B, F, J, N, R and V), cdx2 (C, G K, O, S and W), fxr (D, H, L, P, T and X) genes were detected by qPCR following immunoprecipitation with antibodies against H3K9me1 (A-D), H3K9me2 (E-H), H3K9me3 (I-L), H3K4me2 (M-P), H3K4me3 (Q-T), and normal IgG (U-X). Primers used in qPCR are shown in Additional file 5: Table S2. Each value is the mean ± SEM (n = 8). Distinct letters denote significantly different means, and were determined by one-way analysis of variance and Fisher’s least significant difference test for multiple comparisons (p < 0.05). [file 13578_2016_67_MOESM3_ESM.pdf]

Figure S2

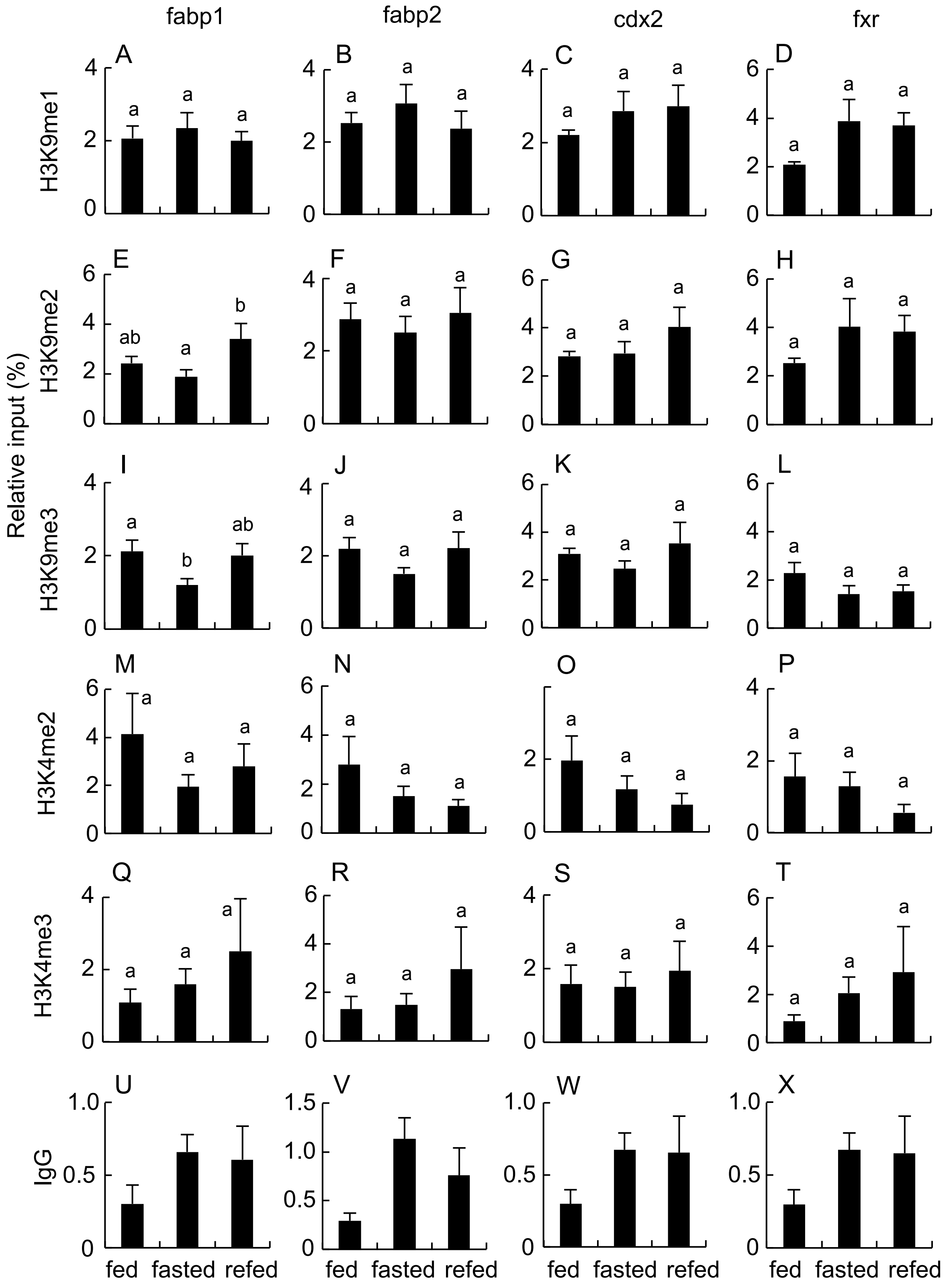

Fig. S2. Epigenetic modifications on fabp1, fabp2, cdx2 and fxr genes in the intestines of fed, fasted and refed *X. laevis*. Chromatin samples were prepared from the intestines from animals that were fed for 22 days (*fed*), fasted for 22 days (*fasted*), and fasted for 21 days and then refed for 1 day (*refed*). Signals of ChIP on fabp1 (*A, E, I, M, Q* and *U*), fabp2 (*B, F, J, N, R* and *V*), cdx2 (*C, G K, O, S* and *W*), fxr (*D, H, L, P, T* and *X*) genes were detected by qPCR following immunoprecipitation with antibodies against H3K9me1 (*A-D*), H3K9me2 (*E-H*), H3K9me3 (*I-L*), H3K4me2 (*M-P*), H3K4me3 (*Q-T*), and normal IgG (*U-X*). Primers used in qPCR are shown in Table S2. Each value is the mean ± SEM ($n = 8$). Distinct letters denote significantly different means, and were determined by one-way analysis of variance and Fisher's least significant difference test for multiple comparisons ($p < 0.05$).
