# Supplementary material for: Morphological, biochemical, transcriptional and epigenetic responses to fasting and refeeding in intestine of Xenopus laevis
Source: Cell Biosci. 2016 Jan 21;6:2. doi: 10.1186/s13578-016-0067-9 (PMC4721045; doi:10.1186/s13578-016-0067-9)
Supplement: Supplementary file 6 — 10.1186/s13578-016-0067-9 The list of primers used and efficiencies in quantitative polymerase chain reaction. [file 13578_2016_67_MOESM6_ESM.docx]

| Table S3. The list of primers used and efficiencies in quantitative polymerase chain reaction. | | | | | | | | | | |
| --- | --- | --- | --- | --- | --- | --- | --- | --- | --- | --- |
| Experiment/category/Gene | Sequence, 5'→3' | Position (nt) | Size amplified (nt) | Tm (°C) | NTC (Cq) | Cq | Slope | R-squared | Efficiency (%) | Accession no. |
| RT-qPCR |  |  |  |  |  |  |  |  |  |  |
| <digestion or absorption> |  |  |  |  |  |  |  |  |  |  |
| fatty acid binding protein 1 (fabp1) | Fwd: TAAAGGGTGTCACCGAGATTG | 20 ~ 40 | 129 | 62 | 32.38 | 16.52-25.63 | 3.5213 | 0.9993 | 92.3 | AF068301 |
|  | Rev: TCTCCCCTGTTGGTGTTTCTA | 148 ~ 128 |  | 62 |  |  |  |  |  |  |
|  |  |  |  |  |  |  |  |  |  |  |
| fatty acid binding protein 2 (fabp2) | Fwd: GGCACATGACAATCTGAAGGT | 146 ~ 166 | 100 | 62 | - | 18.21-25.12 | 3.4618 | 0.9995 | 94.48 | NM_001085877 |
|  | Rev: AGGGTTGTGCAAGGGTAAACT | 245 ~ 225 |  | 62 |  |  |  |  |  |  |
|  |  |  |  |  |  |  |  |  |  |  |
| fatty acid binding protein 6 (fabp6) | Fwd: GGTCGAGAGCCAAGACAACTATG | 132 ~ 154 | 82 | 70 | - | 17.34-23.29 | 3.4323 | 0.9995 | 95.59 | BC099336 |
|  | Rev: AAGTCCCTTCCCTTTTCAATGG | 213 ~ 192 |  | 64 |  |  |  |  |  |  |
|  |  |  |  |  |  |  |  |  |  |  |
| cellular retinol-binding protein type II　(rbp2) | Fwd: TGCAGAAGGGAGAGAAGAACA | 264 ~ 284 | 102 | 62 | - | 18.08-23.70 | 3.5519 | 0.9993 | 91.22 | BC099323 |
|  | Rev: TACACTTGGTGGCACACTTGA | 365 ~ 345 |  | 62 |  |  |  |  |  |  |
|  |  |  |  |  |  |  |  |  |  |  |
| cluster of Differentiation 36 (cd36) | Fwd: GTCCCGTCTACAGGCATTTCTG | 176 ~ 197 | 82 | 68 | - | 23.5-30.29 | 3.36 | 0.997 | 98.44 | PAC:27064799 |
|  | Rev: TGCTCAAGCGCTGGTTTTC | 257 ~ 239 |  | 58 |  |  |  |  |  |  |
|  |  |  |  |  |  |  |  |  |  |  |
| solute carrier family 2 | Fwd: TCATAGCCTCACGACTGGTGAT | 350 ~ 371 | 83 | 66 | - | 24.40-27.44 | 3.5471 | 0.9967 | 91.39 | PAC:27070495 |
| (facilitated glucose/fructose transporter), member 5 (slc2a5) | Rev: TGGAGACATTTCACCCATGTACA | 432 ~ 410 |  | 66 |  |  |  |  |  |  |
|  |  |  |  |  |  |  |  |  |  |  |
| solute carrier family 5 member 1.2 | Fwd: GGAGCATTCTGGGGGTTAATA | 1507 ~ 1527 | 90 | 62 | - | 27.01-31.87 | 3.3115 | 0.9993 | 100.44 | XM_002931895 |
| (sodium/glucose cotransporter), member 1, gene 2 (slc5a1.2) | Rev: TTTCACGCAACTTCCAGTTC | 1596 ~ 1577 |  | 58 |  |  |  |  |  |  |
|  |  |  |  |  |  |  |  |  |  |  |
| solute carrier family 10 | Fwd: CCAAGCAGATGTCGCACAGT | 784 ~ 803 | 76 | 62 | - | 27.24-32.48 | 3.2428 | 0.9999 | 103.41 | PAC:27092916 |
| (sodium/bile acid cotransporter), member 2 (slc10a2) | Rev: AGAGCTGTACAATAGTGGAACAAAGC | 859 ~ 834 |  | 74 |  |  |  |  |  |  |
|  |  |  |  |  |  |  |  |  |  |  |
| peptide transporter 1, pept1 (slc15a1) | Fwd: TCTCCGCAGGGCAACATTAT | 732~ 751 | 77 | 60 | - | 17.18-21.15 | 3.1617 | 0.9942 | 107.15 | PAC:27101690 |
|  | Rev: TGCGATTCCTGTATCTGTTGCT | 808~ 787 |  | 64 |  |  |  |  |  |  |
|  |  |  |  |  |  |  |  |  |  |  |
| solute carrier family 16 | Fwd: GGATACAGTGACACAGCATGGATATC | 354 ~ 379 | 86 | 76 | - | 27.53-31.11 | 3.3959 | 0.9988 | 97 | NM_001087188 |
| (monocarboxylate transporter), member 3 (slc16a3) | Rev: CGATTCACACAGATGCTGCAA | 439 ~ 419 |  | 62 |  |  |  |  |  |  |
|  |  |  |  |  |  |  |  |  |  |  |
| alkaline phosphatase, intestinal, gene 1 (alpi1) | Fwd: GACGACCAAATGTGAATAGCAATC | 1289 ~ 1312 | 59 | 68 | - | 20.15-23.93 | 3.7974 | 0.9861 | 83.37 | NM_001097666 |
|  | Rev: TGCCGCCTGCTGCATA | 1347 ~ 1332 |  | 52 |  |  |  |  |  |  |
|  |  |  |  |  |  |  |  |  |  |  |
| alkaline phosphatase, intestinal, gene 2 (alpi2) | Fwd: CACACCGAGCCAAGAATCTCA | 272 ~ 292 | 82 | 64 | - | 25.53-29.45 | 3.3099 | 0.9998 | 101 | NM_001112847 |
|  | Rev: CCTGATATATGCGAGCTGCAGTTA | 353 ~ 330 |  | 70 |  |  |  |  |  |  |
|  |  |  |  |  |  |  |  |  |  |  |
| alanyl membrane aminopeptidase (anpep) | Fwd: ACAAGCTTATATGCTGATGAATCGTT | 2622 ~ 2647 | 123 | 70 | - | 18.36-23.54 | 3.1553 | 0.9986 | 107.46 | NM_001095122 |
|  | Rev: ATGCATGCTGACAGTCTTTGCT | 2744 ~ 2722 |  | 64 |  |  |  |  |  |  |
|  |  |  |  |  |  |  |  |  |  |  |
| malutase-glucoamylase (mgam) | Fwd: ATCTCTATGGATGGTCACATGCA | 133 ~ 155 | 72 | 66 | - | 18.47-22.90 | 3.3408 | 0.9966 | 99.22 | CB207035 |
|  | Rev: CAATACCTCTTTCCCCAGTAACACTT | 204 ~ 179 |  | 74 |  |  |  |  |  |  |
|  |  |  |  |  |  |  |  |  |  |  |
| neurotensin (nts) | Fwd: TGTTTGATGCTTCTTGCCTTCA | 106 ~ 127 | 80 | 62 | - | 18.64-25.67 | 3.0118 | 1 | 114.8 | NM_001092681 |
|  | Rev: ACATCTGTTTCTAGAGCTTTCATTTCC | 185 ~ 159 |  | 74 |  |  |  |  |  |  |
|  |  |  |  |  |  |  |  |  |  |  |
| vasoactive intestinal peptide (vip) | Fwd: CCACCATTTTCAGACAACTATGATG | 758 ~ 782 | 81 | 70 | 37.7 | 25.69-30.08 | 3.1508 | 0.9997 | 107.67 | NM_001085714 |
|  | Rev: GGGTATCTTTCAAAGGTTCAATTGG | 838 ~ 814 |  | 70 |  |  |  |  |  |  |
|  |  |  |  |  |  |  |  |  |  |  |
| somatostatin (sst) | Fwd: GCCAGTTCCTGCAGAAATCAC | 168 ~ 188 | 74 | 64 | 31.39 | 23.18-26.38 | 3.2207 | 0.9997 | 104.41 | NM_001089250 |
|  | Rev: TAGCAACTCTGCCAGGAAATACTTG | 241 ~ 217 |  | 72 |  |  |  |  |  |  |
|  |  |  |  |  |  |  |  |  |  |  |
| villin 1 (vil1) | Fwd: CACATACAATGTAAAGCGCCTTCT | 491 ~ 514 | 106 | 68 | 35.85 | 19.02-24.95 | 3.065 | 0.9952 | 111.97 | NM_001087034 |
|  | Rev: CAGGAAGACATCACCTAGATTAAAGCT | 596 ~ 570 |  | 76 |  |  |  |  |  |  |
|  |  |  |  |  |  |  |  |  |  |  |
| glucagon-like peptide 1 (glp1) | Fwd: CAGAGGGAACATTCACTAATG | 605 ~ 625 | 72 | 60 | - | 25.72-29.97 | 3.0531 | 0.9769 | 112.59 | NM_001085673 |
|  | Rev: GCCAGCCAACAAATTCTTTG | 676 ~ 657 |  | 58 |  |  |  |  |  |  |
|  |  |  |  |  |  |  |  |  |  |  |
| glucagon-like peptide 1 receptor (glp1r) | Fwd: TGCACTTGCGGCTTAGCA | 736 ~ 753 | 68 | 56 | - | 27.31-29.94 | 3.2762 | 0.9913 | 101.94 | BC170485 |
|  | Rev: CCCGTACTGCATGAGGACTGT | 803 ~ 783 |  | 66 |  |  |  |  |  |  |
|  |  |  |  |  |  |  |  |  |  |  |
| gastric inhibitory polypeptide (gip) | Fwd: GAGATACTCGGAGGCCATCCT | 169 ~ 189 | 79 | 66 | 38.37 | 23.37-27.69 | 3.0462 | 0.999 | 113 | NM_001097117 |
|  | Rev: CCAGTCGACAAAGTTCTTCTTGAG | 224 ~ 247 |  | 70 |  |  |  |  |  |  |
| <apoptosis> |  |  |  |  |  |  |  |  |  |  |
| caspase 1, apoptosis-related cysteine peptidase (casp1) | Fwd:TTAGGATACCAAGTCCATCCACATAC | 571 ~ 596 | 83 | 74 | 39.3 | 24.98-30.86 | 3.2025 | 0.995 | 105.24 | NM_001087754 |
|  | Rev: TCCTCCTGGGCAGCAAAC | 653 ~ 636 |  | 58 |  |  |  |  |  |  |
|  |  |  |  |  |  |  |  |  |  |  |
| caspase 3, apoptosis-related cysteine peptidase (casp3) | Fwd: CAGAGGAGACCGATGCAAGAC | 467 ~ 487 | 65 | 66 | - | 23.32-28.25 | 3.3047 | 0.9994 | 100.72 | NM_001087756 |
|  | Rev: CCCTGCATGCCTGGAT | 531 ~ 515 |  | 52 |  |  |  |  |  |  |
|  |  |  |  |  |  |  |  |  |  |  |
| caspase 7, apoptosis-related cysteine peptidase (casp7) | Fwd: TCTTTGCAACGTCCTTAACGAA | 785 ~ 806 | 63 | 62 | - | 25.20-29.18 | 3.8467 | 0.9952 | 81.95 | NM_001097803 |
|  | Rev: ACTCTGGTCAGGATCTGCATGA | 847 ~ 826 |  | 66 |  |  |  |  |  |  |
|  |  |  |  |  |  |  |  |  |  |  |
| caspase 8, apoptosis-related cysteine peptidase (casp8) | Fwd: ATTGCCAACCCCAACAACAC | 716 ~ 735 | 80 | 60 | - | 27.04-31.07 | 3.5128 | 0.998 | 92.61 | NM_001085565 |
|  | Rev: GACCACACACCAGCCATGAG | 795 ~ 776 |  | 64 |  |  |  |  |  |  |
|  |  |  |  |  |  |  |  |  |  |  |
| caspase 9 , apoptosis-related cysteine peptidase (casp9) | Fwd: CTGCTGCCTGGTGGTGATC | 639 ~ 657 | 67 | 62 | - | 24.72-29.28 | 3.6747 | 0.9965 | 87.12 | NM_001085566 |
|  | Rev: AACCCCTCCTGGGAACTGAAT | 705 ~ 685 |  | 64 |  |  |  |  |  |  |
|  |  |  |  |  |  |  |  |  |  |  |
| nitric oxide synthase 1 (nos1) | Fwd: GCAACCTCAGATCAGCAATCAC | 1756 ~ 1777 | 87 | 66 | - | 25.06-30.80 | 3.8439 | 0.9834 | 82.03 | NM_001085686 |
|  | Rev: AGCGAATAAGCTGAGCATTCCA | 1842 ~ 1821 |  | 64 |  |  |  |  |  |  |
|  |  |  |  |  |  |  |  |  |  |  |
| nitric oxide synthase 2 (nos2) | Fwd: CGGTGCATTGGACGAATACA | 238 ~ 257 | 80 | 60 | 35.96 | 24.50-30.60 | 3.3181 | 0.997 | 100.16 | PAC:27059000 |
|  | Rev: TTAAACATTTCCTTGGCTGTCTTG | 317 ~ 294 |  | 66 |  |  |  |  |  |  |
|  |  |  |  |  |  |  |  |  |  |  |
| <proliferation> |  |  |  |  |  |  |  |  |  |  |
| mechanistic target of rapamycin (mtor) | Fwd: AGCTGCCTCAGCTCACTTCTTT | 398 ~ 419 | 71 | 66 | - | 24.11-30.30 | 3.5841 | 0.9946 | 90.11 | PAC:27082461 |
|  | Rev: CTCCAAATCTCGGCACATAAGTAA | 468 ~ 445 |  | 68 |  |  |  |  |  |  |
|  |  |  |  |  |  |  |  |  |  |  |
| mtor associated protein (mlst8) | Fwd: GTCAATGCTCCAATCAACTGTGTT | 458 ~ 481 | 82 | 68 | 36.97 | 27.12-30.42 | 3.824 | 0.9997 | 82.6 | NM_001089913 |
|  | Rev: TGTGAATGGCTCCACTCTGATC | 539 ~ 518 |  | 66 |  |  |  |  |  |  |
|  |  |  |  |  |  |  |  |  |  |  |
| regulatory associated protein of mtor, complex 1 (raptor) | Fwd: CTGTTGATAGTTCTTGCCAAGCA | 1504 ~ 1526 | 94 | 66 | - | 26.54-31.12 | 3.5657 | 0.999 | 90.74 | NM_001094708 |
|  | Rev: TGCTCGGCCGGCATATAT | 1597 ~ 1580 |  | 56 |  |  |  |  |  |  |
|  |  |  |  |  |  |  |  |  |  |  |
| rptor independent companion of mtor, complex 2 (rictor) | Fwd: AGAGGAGAATGTGATCCCTGATATTG | 2523 ~ 2548 | 81 | 74 | 35.51 | 26.27-29.53 | 3.5597 | 0.9966 | 91 | PAC:27062595 |
|  | Rev: ACATACGCACACGTCCCTCTTAT | 2603 ~ 2581 |  | 68 |  |  |  |  |  |  |
|  |  |  |  |  |  |  |  |  |  |  |
| mitogen-activated protein kinase associated protein 1 (mapkap1) | Fwd: GACTGATTTGCTGGCAGTATACGA | 861 ~ 884 | 82 | 70 | - | 25.00-27.61 | 3.469 | 0.9986 | 94.21 | NM_001086997 |
|  | Rev: GCTATGTGCAAGCAGAATGCA | 942 ~ 922 |  | 62 |  |  |  |  |  |  |
|  |  |  |  |  |  |  |  |  |  |  |
| ataxia telangiectasia mutated (atm) | Fwd: TCGATCTGTTGCACCGAGTCT | 4381 ~ 4401 | 139 | 64 | 36.95 | 24.33-28.41 | 3.5997 | 0.9982 | 89.58 | NM_001088499 |
|  | Rev: AAGTCGCACACCTTTCTTGAAT | 4519 ~ 4497 |  | 62 |  |  |  |  |  |  |
|  |  |  |  |  |  |  |  |  |  |  |
| marker of proliferation Ki-67 (mki67) | Fwd: GCTGCCACATGTCTCCAAAGA | 164 ~ 185 | 81 | 64 | - | 24.17-28.48 | 3.2343 | 0.9949 | 103.79 | NM_001135081 |
|  | Rev: CGGCACTTATGTTGATGACAAAG | 244 ~ 222 |  | 66 |  |  |  |  |  |  |
|  |  |  |  |  |  |  |  |  |  |  |
| proliferating cell nuclear antigen (pcna) | Fwd: CAGCCGCCGCCATGT | 28 ~ 42 | 60 | 52 | 37.44 | 24.13-29.20 | 3.5766 | 0.9931 | 90.37 | NM_001087542 |
|  | Rev: CCAACACCTTCTTCAGGATGGA | 87 ~ 66 |  | 66 |  |  |  |  |  |  |
| <regulation of gene expression> |  |  |  |  |  |  |  |  |  |  |
| farnesoid X receptor (fxr) | Fwd: TGGGATCCCTTAATGTGACTG | 1294 ~ 1314 | 84 | 62 | - | 26.76-32.29 | 3.6326 | 0.9853 | 88.49 | NM_001088774 |
|  | Rev: TTTGTAAGAGTGGGCGATCTG | 1377 ~ 1357 |  | 62 |  |  |  |  |  |  |
|  |  |  |  |  |  |  |  |  |  |  |
| caudal type homeobox 1 (cdx1) | Fwd: CCTGGGATTAACAGTGATCCA | 256~ 276 | 138 | 62 | 33.06 | 21.66-24.83 | 3.5117 | 0.9982 | 92.65 | NM_001087706 |
|  | Rev: ACTGAAAGCGATCTGTGTTGG | 393 ~ 373 |  | 62 |  |  |  |  |  |  |
|  |  |  |  |  |  |  |  |  |  |  |
| caudal type homeobox 2 (cdx2) | Fwd: GCGGCATCACTACGAATGG | 530 ~ 548 | 60 | 60 | - | 23.20-26.48 | 3.3843 | 0.9982 | 97.46 | NM_001096486 |
|  | Rev: TTGGATCCCGGTATGTTTGC | 589 ~ 570 |  | 60 |  |  |  |  |  |  |
|  |  |  |  |  |  |  |  |  |  |  |
| cAMP responsive element binding protein 1 (creb1) | Fwd: GAGGCAGTGTAACCCGGTAACT | 88 ~ 109 | 70 | 68 | - | 23.95-29.76 | 3.4228 | 0.9992 | 95.96 | NM_001086603 |
|  | Rev: CTGTGACGGCATCTCCACTCT | 157~ 137 |  | 66 |  |  |  |  |  |  |
|  |  |  |  |  |  |  |  |  |  |  |
| liver X receptor alpha (lxra) | Fwd: AGCTCACACCTCAGCAAGAAA | 799 ~ 819 | 139 | 62 | 35.7 | 24.66-27.91 | 3.8079 | 0.9954 | 83.07 | NM_001092614 |
|  | Rev: TGTCGAGCTTCTCTGCTGTTT | 937 ~ 917 |  | 62 |  |  |  |  |  |  |
|  |  |  |  |  |  |  |  |  |  |  |
| thyroid hormone receptor beta (thrb) | Fwd: GCCATGTGAAGACCAGATCAT | 624 ~ 644 | 172 | 62 | - | 26.03-30.00 | 3.133 | 0.9998 | 108.54 | NM_001096713 |
|  | Rev: GTCACTGCCATCTCACCATTT | 796 ~ 776 |  | 62 |  |  |  |  |  |  |
|  |  |  |  |  |  |  |  |  |  |  |
| peroxisome proliferator-activated receptor gamma, | Fwd: ATCTTCTGTGCCACCTGCTAA | 920 ~ 940 | 104 | 62 | - | 27.04-29.69 | 3.8524 | 0.9924 | 81.79 | FJ710600 |
| coactivator 1 alpha (ppargc1a) | Rev: GTGCGTACAGTTCTGATTGC | 1023 ~ 1004 |  | 60 |  |  |  |  |  |  |
|  |  |  |  |  |  |  |  |  |  |  |
| nuclear receptor subfamily 3 group C member 1 (nr3c1) | Fwd: TCAAAACTGGCAGCGCTTT | 2228 ~ 2246 | 68 | 56 | 38.66 | 22.67-25.71 | 3.4303 | 0.9992 | 95.67 | NM_001088062 |
|  | Rev: GATTCTCAGCCACCTCATGCA | 2295 ~ 2275 |  | 64 |  |  |  |  |  |  |
|  |  |  |  |  |  |  |  |  |  |  |
| hepatocyte nuclear factor homeobox A (hnf1a) | Fwd: TATCTGCAGCAGCACAACATC | 537 ~ 557 | 105 | 62 | - | 22.26-25.47 | 3.7388 | 0.9998 | 85.13 | NM_001101744 |
|  | Rev: TACAGGGCTGCTCTCTTTTGA | 661 ~ 641 |  | 62 |  |  |  |  |  |  |
|  |  |  |  |  |  |  |  |  |  |  |
| hepatocyte nuclear factor homeobox B (hnf1b) | Fwd: CATGGATCACCACACCATACA | 1124 ~ 1144 | 141 | 62 | - | 32.56-37.58 | 3.4485 | 0.9971 | 94.98 | NM_001089811 |
|  | Rev: GCTGGTCACCATAGCATTGTT | 1264~ 1244 |  | 62 |  |  |  |  |  |  |
|  |  |  |  |  |  |  |  |  |  |  |
| hepatocyte nuclear factor 4 alpha (hnf4a) | Fwd: GCAAAGTATATCCCGGCTTTC | 699~ 719 | 104 | 62 | 34.42 | 23.08-27.31 | 3.3555 | 0.9986 | 98.62 | NM_001086601 |
|  | Rev: ACCTCTTTGTAGCCCCAAGAA | 802 ~ 782 |  | 62 |  |  |  |  |  |  |
|  |  |  |  |  |  |  |  |  |  |  |
| peroxisome proliferator-activated receptor alpha (ppara) | Fwd: GGAAACAGCCCTGGGTCAA | 495 ~ 513 | 67 | 60 | - | 24.63-27.22 | 3.916 | 0.9991 | 80.04 | NM_001095362 |
|  | Rev: TCGACGCAGGAGATAAAGTGTCT | 561 ~ 539 |  | 68 |  |  |  |  |  |  |
|  |  |  |  |  |  |  |  |  |  |  |
| peroxisome proliferator-activated receptor delta (ppard) | Fwd: GAGGCTATCTTTTGCATGCTG | 801 ~ 821 | 190 | 62 | 36.87 | 26.35-29.25 | 3.2601 | 0.9993 | 102.65 | NM_001087841 |
|  | Rev: CAGGAACTCACGGGTAACAAA | 990 ~ 970 |  | 62 |  |  |  |  |  |  |
|  |  |  |  |  |  |  |  |  |  |  |
| retinoic acid receptor alpha (rara) | Fwd: GCGAGTTTCTCTGGACATTGA | 1185 ~ 1205 | 140 | 62 | - | 23.90-27.19 | 3.5115 | 0.9991 | 92.65 | NM_001090254 |
|  | Rev: ATTTCAGGAGGGTGATCTGGT | 1324 ~ 1304 |  | 62 |  |  |  |  |  |  |
|  |  |  |  |  |  |  |  |  |  |  |
| retinoic acid receptor beta (rarb) | Fwd: TACCCTCAACCGAACTCAGA | 639 ~ 658 | 112 | 60 | 36.7 | 25.20-30.23 | 3.5037 | 0.9992 | 92.94 | DQ465004 |
|  | Rev: GCCAGTTTCTGTGTCATCCAT | 750 ~ 730 |  | 62 |  |  |  |  |  |  |
|  |  |  |  |  |  |  |  |  |  |  |
| retinoic acid receptor gamma (rarg) | Fwd: CTATGAGATGCCCCCAGAAAT | 656 ~ 681 | 102 | 62 | - | 24.92-29.57 | 3.2772 | 0.9968 | 101.9 | NM_001088194 |
|  | Rev: TTCGTGGTGTATTTGCCTAGC | 757 ~ 737 |  | 62 |  |  |  |  |  |  |
|  |  |  |  |  |  |  |  |  |  |  |
| retinoid X receptor alpha (rxra) | Fwd: AGTTGGACTGCTGGATACGATTTT | 154 ~ 177 | 64 | 68 | - | 23.83-27.82 | 3.7819 | 0.9986 | 83.83 | L11446 |
|  | Rev: GGCCCGAGGAGCTTAAGG | 217 ~ 200 |  | 60 |  |  |  |  |  |  |
|  |  |  |  |  |  |  |  |  |  |  |
| retinoid X receptor beta (rxrb) | Fwd: GCAGATATCAGAAATGCCTTGCT | 561 ~ 583 | 65 | 66 | - | 24.97-30.13 | 3.4417 | 0.9841 | 95.23 | NM_001087467 |
|  | Rev: CCGCTGACGCTCCTCTTG | 625 ~ 608 |  | 60 |  |  |  |  |  |  |
|  |  |  |  |  |  |  |  |  |  |  |
| retinoid X receptor gamma (rxrg) | Fwd: CCAGTATTGCCGGTATCAGAA | 675 ~ 695 | 136 | 62 | - | 30.94-33.95 | 3.308 | 0.9986 | 100.6 | NM_001095479 |
|  | Rev: CACTGGCATTTCCTCACTTGT | 810 ~ 790 |  | 62 |  |  |  |  |  |  |
| <metabolism> |  |  |  |  |  |  |  |  |  |  |
| hexokinase 1 (hk1) | Fwd: GGACATTATCCATGGTAGTGGTACTAGA | 435 ~ 462 | 84 | 80 | - | 28.03-32.07 | 3.21 | 0.998 | 104.9 | NM_001103186 |
|  | Rev: TTGATCTGTTTCTTCTGCATGAAGT | 518 ~ 494 |  | 68 |  |  |  |  |  |  |
|  |  |  |  |  |  |  |  |  |  |  |
| pyruvate kinase, liver and RBC (pklr) | Fwd: GAGATGATAAAGGCTGGGATGAAC | 197 ~ 220 | 81 | 70 | - | 24.49-27.48 | 3.2583 | 0.9989 | 102.73 | NM_001090045 |
|  | Rev: GTGAATAGACCCAGCGTGGTACTC | 277 ~ 254 |  | 74 |  |  |  |  |  |  |
|  |  |  |  |  |  |  |  |  |  |  |
| phosphofructokinase, muscle (pkfm) | Fwd: AAGTCACCAGAGGACCTTTGTTCT | 759 ~ 782 | 82 | 70 | - | 28.30-29.51 | 3.4933 | 0.9999 | 93.31 | NM_001093452 |
|  | Rev: ACCACATGCAAGGGCTGTTAC | 840 ~ 820 |  | 64 |  |  |  |  |  |  |
|  |  |  |  |  |  |  |  |  |  |  |
| pyruvate dehydrogenase kinase 4 (pdk4) | Fwd: CAAGGTAGAAAGCTTCTCGGAAGAC | 1524 ~ 1547 | 82 | 74 | - | 15.00-20.18 | 3.1621 | 0.9983 | 107.13 | NM_001086628 |
|  | Rev: GGTAGTGGTCCTGGGAGAAAGG | 1605 ~ 1584 |  | 70 |  |  |  |  |  |  |
|  |  |  |  |  |  |  |  |  |  |  |
| phospoenolypyuvate carboxykinase1 (pck1) | Fwd: GCAGCTGAACATAAAGGCAAG | 1498 ~ 1521 | 93 | 62 | - | 25.16-28.77 | 3.5056 | 0.9974 | 92.87 | NM_001086477 |
|  | Rev: TGAGCCAGTGAGCAAGGTATT | 1590~ 1570 |  | 62 |  |  |  |  |  |  |
|  |  |  |  |  |  |  |  |  |  |  |
| fructose-1,6-bisphosphatase 1 (fbp1) | Fwd: ACATTGACTGCCTGGCTTCTA | 445~ 465 | 126 | 62 | - | 22.59-27.07 | 3.4059 | 0.9982 | 96.61 | NM_001087059 |
|  | Rev: ATAGAGCATAGCCTGCTGCAA | 570 ~ 550 |  | 62 |  |  |  |  |  |  |
|  |  |  |  |  |  |  |  |  |  |  |
| fibroblast growth factor 19 (fgf19) | Fwd: TACCTCCGCATACACGAGGAT | 178 ~ 198 | 84 | 64 | 38.75 | 22.71-29.11 | 3.0804 | 0.9987 | 111.17 | PAC:27092531 |
|  | Rev: TGCAGTCGCTCTGATTTCCA | 261 ~ 242 |  | 60 |  |  |  |  |  |  |
|  |  |  |  |  |  |  |  |  |  |  |
| glucose-6-phosphatase, catalytic subunit (g6pc) | Fwd: TGCTGGCCTCCTTAGAAACTT | 806 ~ 826 | 104 | 62 | - | 26.72-34.21 | 3.2689 | 0.9975 | 102.26 | NM_001086134 |
|  | Rev: TGAACTGTGCACCCTTCTTTC | 909 ~ 889 |  | 62 |  |  |  |  |  |  |
|  |  |  |  |  |  |  |  |  |  |  |
| glucose-6-phosphatase, catalytic 2 (g6pc2) | Fwd: AAGGCGAGCCTGAAGAAATAC | 649 ~ 669 | 109 | 62 | - | 24.14-27.40 | 3.3197 | 0.9987 | 100.09 | NM_001094213 |
|  | Rev: TCCAGAGTCCAAAGGAGATCA | 758 ~ 738 |  | 62 |  |  |  |  |  |  |
|  |  |  |  |  |  |  |  |  |  |  |
| glutamate-ammonia ligase (glul) | Fwd: AGTCTTGAAATACAACCGCAAGTCT | 367 ~ 369 | 75 | 70 | - | 21.40-25.13 | 3.4728 | 0.9971 | 94.07 | NM_001092398 |
|  | Rev: CCCACCATCTCCATGATCTTCT | 441 ~ 420 |  | 66 |  |  |  |  |  |  |
|  |  |  |  |  |  |  |  |  |  |  |
| 3-hydroxy-3-methylglutaryl-CoA reductase (hmgcr) | Fwd: CAGAGGTTGCAGAGCCATAATG | 1800 ~ 1821 | 65 | 66 | - | 23.31-25.60 | 3.5152 | 0.9981 | 92.52 | NM_001087811 |
|  | Rev: TCATCCCATCAGCCAGAACAC | 1864 ~ 1844 |  | 64 |  |  |  |  |  |  |
|  |  |  |  |  |  |  |  |  |  |  |
| apolipoprotein A-I (apoa1) | Fwd: ACGCCCTTCGCTCAAGACT | 568 ~ 586 | 62 | 60 | - | 15.85-21.17 | 3.4922 | 0.9995 | 93.35 | PAC:27078607 |
|  | Rev: TTTCGCAACCAGCTTTTGC | 629 ~ 611 |  | 56 |  |  |  |  |  |  |
|  |  |  |  |  |  |  |  |  |  |  |
| hydroxyacyl-CoA dehydrogenase/3-ketoacyl-CoA thiolase | Fwd: CAGGAGGCCGGCTATCTTG | 985 ~ 1003 | 74 | 62 | - | 22.35-27.20 | 3.5086 | 1 | 92.76 | NM_001092149 |
| /enoyl-CoA hydratase, alpha subunit (hadha) | Rev: ATTAGTGCTTTGGACTCTGAGGTCAT | 1058 ~ 1033 |  | 74 |  |  |  |  |  |  |
|  |  |  |  |  |  |  |  |  |  |  |
| acyl-CoA oxidase 1 (acox1) | Fwd: ACATGGGATCAGCAAGAACAC | 1705 ~ 1725 | 132 | 62 | - | 22.60-25.60 | 3.426 | 0.9999 | 95.83 | NM_001096680 |
|  | Rev: CCACTGCATTAGGACGGATTA | 1826 ~ 1806 |  | 62 |  |  |  |  |  |  |
|  |  |  |  |  |  |  |  |  |  |  |
| acyl-CoA oxidase 2 (acox2) | Fwd: GTACCAAACCCAGCAACAGAA | 1069 ~ 1089 | 138 | 62 | 33.54 | 23.52-28.51 | 3.5458 | 0.9984 | 91.44 | NM_001091064 |
|  | Rev: CAGGAAGGGAGTCAAAATTCC | 1206~ 1186 |  | 62 |  |  |  |  |  |  |
|  |  |  |  |  |  |  |  |  |  |  |
| acyl-CoA dehydrogenase very long chain (acadvl) | Fwd: GGCAGCAGCTCAGGAATCTC | 225 ~ 244 | 71 | 64 | 38.05 | 26.16-31.08 | 3.5319 | 0.9986 | 91.93 | PAC:27074862 |
|  | Rev: AAACCTGCTCGGAGTGGATCT | 295 ~ 275 |  | 64 |  |  |  |  |  |  |
|  |  |  |  |  |  |  |  |  |  |  |
| glucose-6-phosphate dehydrogenase (g6pd) | Fwd: GGAGATCTGGCCAAAAAGAAGA | 247 ~ 268 | 68 | 64 | - | 22.54-27.04 | 3.2187 | 0.999 | 104.5 | NM_001086550 |
|  | Rev: TCTGGGAGCAAACCATCGTT | 314 ~ 295 |  | 60 |  |  |  |  |  |  |
|  |  |  |  |  |  |  |  |  |  |  |
| <others> |  |  |  |  |  |  |  |  |  |  |
| mex-3 RNA binding family member A (mex3a) | Fwd: GAGCCAAAACTAACACCTACATCAAG | 120 ~ 145 | 70 | 74 | - | 26.39-30.26 | 3.9169 | 0.9963 | 80.01 | BC130195 |
|  | Rev: CGACCCGTCACCATGAACA | 189 ~ 171 |  | 60 |  |  |  |  |  |  |
|  |  |  |  |  |  |  |  |  |  |  |
| leucine-rich repeat containing G protein-coupled receptor 5 (lgr5) | Fwd: TTTATAGACAACCCTATTCAACATGTG | 999 ~ 1024 | 87 | 72 | 36.24 | 27.37-31.16 | 3.7393 | 0.9991 | 85.11 | NM_001199223 |
|  | Rev: GTGAAGCCCCATTAAGAATCAAAG | 1085 ~ 1062 |  | 68 |  |  |  |  |  |  |
|  |  |  |  |  |  |  |  |  |  |  |
| ribosomal protein L8 (rpl8) | Fwd: CAAGGCAAAGAGAAACTGCTG | 625 ~ 645 | 98 | 62 | - | 22.31-24.77 | 3.496 | 0.9976 | 93.22 | NM_001086996 |
|  | Rev: GCTTACCAATGTGTTGGTGGT | 722 ~ 702 |  | 62 |  |  |  |  |  |  |
|  |  |  |  |  |  |  |  |  |  |  |
| RT-qPCR for pre-mRNA (intron specific primer) |  |  |  |  |  |  |  |  |  |  |
| ribosomal protein L8 (rpl8) | Fwd: TGAGGCCTGTGTTGGTATCTG | 5024 ~ 5044 | 149 | 64 | - | 30.30-33.56 | 3.3175 | 0.8769 | 100.19 | PAC:31620599 |
|  | Rev: TTCTGAACACCAATCTCTTCTGC | 5172 ~ 5150 |  | 66 |  |  |  |  |  |  |
|  |  |  |  |  |  |  |  |  |  |  |
| fatty acid binding protein 1 (fabp1) | Fwd: GTGAGTGTTGCCATTCAG | 2197 ~ 2214 | 102 | 54 | - | 27.48-34.59 | 3.7331 | 0.9762 | 85.3 | PAC:27102411 |
|  | Rev: CTGCAAGGTGTAAAGAAGAG | 2298 ~ 2279 |  | 58 |  |  |  |  |  |  |
|  |  |  |  |  |  |  |  |  |  |  |
| fatty acid binding protein 2 (fabp2) | Fwd: CCTTCGGCACTCGGGTTA | 1701 ~ 1718 | 67 | 58 | - | 25.28-30.06 | 3.4058 | 0.9935 | 96.62 | PAC:27069435 |
|  | Rev: TGGCCACGCTGAGAGATATG | 1767 ~ 1748 |  | 62 |  |  |  |  |  |  |
|  |  |  |  |  |  |  |  |  |  |  |
| caudal type homeobox 2 (cdx2) | Fwd: GTCAGATAGGAGCAGGGTGATGT | 2997 ~ 3019 | 54 | 70 | 35.68* | 28.07-33.11 | 3.5979 | 0.9858 | 89.64 | PAC:27107320 |
|  | Rev: AGGACAGGGCGCCTGACT | 3050 ~ 3033 |  | 60 |  |  |  |  |  |  |
|  |  |  |  |  |  |  |  |  |  |  |
| farnesoid X receptor (fxr) | Fwd: CGCCTGAAGTTGCCTAATGAG | 339 ~ 359 | 101 | 64 | 36.45* | 29.96-34.69 | 3.3949 | 0.9932 | 97.04 | PAC:27083150 |
|  | Rev: TTCCCGCTGGCTAGAATGTG | 439 ~ 418 |  | 62 |  |  |  |  |  |  |
|  |  |  |  |  |  |  |  |  |  |  |
| ChIP assay |  |  |  |  |  |  |  |  |  |  |
| fatty acid binding protein 1 (fabp1) | Fwd: GCTCCAAAGTCTTACGCAATGA | 80 ~ 101 | 67 | 64 | 36.18* | 27.99-35.69 | 3.2868 | 0.9962 | 101.49 | AF068301 |
|  | Rev: TCCCCTGTTGGTGTTTCTAGCT | 146 ~ 125 |  | 66 |  |  |  |  |  |  |
|  |  |  |  |  |  |  |  |  |  |  |
| fatty acid binding protein 2 (fabp2) | Fwd: GCACATGACAATCTGAAGGTCATAA | 137 ~ 159 | 82 | 70 | - | 26.70-35.93 | 3.359 | 0.9982 | 98.48 | NM_001085877 |
|  | Rev: CAATGTTACGGAATGTGCTGGAT | 218 ~ 196 |  | 66 |  |  |  |  |  |  |
|  |  |  |  |  |  |  |  |  |  |  |
| caudal type homeobox 2 (cdx2) | Fwd: GGCATCTGAGCTGGCAGTTAG | 86 ~ 106 | 87 | 66 | - | 27.45-36.22 | 3.1783 | 0.9972 | 106.36 | NM_001096486 |
|  | Rev: CCAACAAGTAACTCACGTACATGGT | 172 ~ 148 |  | 72 |  |  |  |  |  |  |
|  |  |  |  |  |  |  |  |  |  |  |
| farnesoid X receptor (fxr) | Fwd: GGGAATTCGTGTTAATGAGACAGAG | 43 ~ 67 | 88 | 72 | 32.28* | 30.88-36.79 | 3.2752 | 0.9973 | 101.99 | NM_001088774 |
|  | Rev: GCAAGACAGTATGCATCAGAAACTG | 130 ~ 106 |  | 72 |  |  |  |  |  |  |
| ribosomal protein l8 (rpl8) | Fwd: GAGAAAAGGTGCAGGCTCTGTT | 81 ~ 102 | 75 | 66 | - | 24.77-34.94 | 3.1096 | 0.9522 | 109.69 | NM_001086996 |
|  | Rev: CGATAGCCCGAAGCTTAGCA | 155 ~ 136 |  | 62 |  |  |  |  |  |  |
| *Dissociation analyses showed different peaks between the results from non-template control and target samples, suggesting that the product amplified from non-template control was different from that from target samples. | | | | | | | | | | |
