# Supplementary material for: Morphological, biochemical, transcriptional and epigenetic responses to fasting and refeeding in intestine of Xenopus laevis
Source: Cell Biosci. 2016 Jan 21;6:2. doi: 10.1186/s13578-016-0067-9 (PMC4721045; doi:10.1186/s13578-016-0067-9)
Supplement: Supplementary file 7 — 10.1186/s13578-016-0067-9 Detailed qPCR information. [file 13578_2016_67_MOESM7_ESM.docx]

Table S4. Detailed qPCR information.

A. RT- qPCR information

**Animals**

Description *Xenopus laevis* 1 years old (from Watanabe Breeding, Hyogo, Japan)

**Experimental design**

Definition of experimental and control groups Fed group: Fed 8 crickets/frog every day (22 days).

Fasted group: Fasted in experiment term (22 days).

Refed group: Fasted 21 days and fed 8 crickets/frog only last day.

Number within each group n = 8

**Nucleic acid extraction**

Procedure and/or instrumentation RNA extraction: the AGPC method (Chomczynski and sacchi, 1987)

Details of DNase or RNase treatment After adding 1 U/μL RQ1 DNase, incubation for 30 min at 37 °C (Roche, Tokyo, Japan).

Contamination assessment (DNA or RNA) The Cqs of DNase-treated samples were slightly higher than those of untreated samples.

Nucleic acid quantification Concentrations, 200-500 μg/mL; volume, 100 μL

Instrument and method Instrument, BioSpec-nano (SHIMADZU, Kyoto, Japan); Method, Instruction manual

Purity (A260/A280) A_260_/A_280_: 1.90-2.10

RNA integrity: method/instrument 28S rRNA and 18S rRNA bands were electrophoretically detected.

Method Electrophoresis in a 1% agarose gel containing 2 M formaldehyde

**Reverse transcription**

Complete reaction conditions Final concentrations of reaction reagents:

20 ng/μL RNA sample,

1 x Taqman RT buffer,

5.5 mM MgCl_2_,

500 μM each dNTP,

2.5 μM Random hexamers,

0.4 U/μL RNase inhibitor,

1.25 U/μL MultiScribe reverse transcriptase

Amount of RNA and reaction volume Amount of RNA, 200 ng; reaction volume, 10 μL

Priming oligonucleotide (if using GSP) and concentration Random hexamers; final concentration, 2.5 μM

Reverse transcriptase and concentration MultiScribe reverse transcriptase; final concentration, 1.25 U/μL

Temperature and time 25°Cfor 10 min, 48°C for 30 min, and then 95°C for 5 min

Manufacturer of reagents and catalogue numbers Taqman RT reagent kit

Applied Biosystems (Foster City, CA, USA), Cat. No. N8080234

Cqs with and without reverse transcription Results of DNase treated samples.

Storage conditions of cDNA -20°C

**qPCR target information**

Gene symbol See the text and Table. S2

Sequence accession number See Table S2

Location of amplicon See Table S2

Amplicon length See Table S2

In silico specificity screen (BLAST, and so on) Theprimer specificitywas confirmed using BLAST search.

**PCR oligonucleotides**

Primer sequences See Table S2

**qPCR protocol**

Complete reaction conditions Final volume of reaction reagents:

12.5 μL 2 x Power SYBR Green Master Mix,

2 μL 2.5 μM each Primer,

8.5 μL Diethylpyrocarbonate-treated water,

2 μL cDNA mixture (corresponding to 40 ng RNA)

Reaction volume and amount of cDNA/DNA Reaction volume, 25 μL; cDNA mixture after reverse transcription, 2 μL

Primer, (probe), Mg^2+^, and dNTP concentrations Primers, each 200 nM (final conc.)

Power SYBR Green Master Mix includes Mg^2+^ and dNTP, whose concentrations are unclear.

Polymerase identity and concentration Power SYBR Green Master Mix includes Ampli Taq Gold DNA Polymerase, whose concentration is unclear.

Buffer/kit identity and manufacturer Power SYBR Green Master Mix, Applied Biosystems (Foster City, CA, USA), Cat. No. 4367659

Exact chemical composition of the buffer Unclear

Additives (SYBR Green I, DMSO, and so forth) Power SYBR Green Master Mix includes SYBR Green I and Passive reference, whose concentrations are unclear.

Manufacturer of plates/tubes and catalog number ABgene PCR Detection plates (Thermo Scientific, Yokohama, Japan),

Cat. No. AB-1100

Complete thermocycling parameters 50°C for 2 min and 95°C for 10 min, and 40 cycles of 95°C for 15 s, 60°C for 1 min

Manufacturer of qPCR instrument ABI Prism 7000 Sequence Detection System

(Applied Biosystems, Foster City, CA, USA)

**Data analysis**

qPCR analysis program (source, version) ABI PRISM 7000 SDS software version 1.0 (build 81 rev3)

Method of Cq determination Following the instruction manual,

Cqs were arbitrarily determined the appropriate position.

Outlier identification and disposition None

Justification of number and choice of reference genes Two genes rpl8 and eef1a1 were tested.

The variation in Cqs of rpl8 was less than that of eef1a1 among the groups.

Description of normalization method The 2^-ΔΔCq^ method (Livak and Schmittgen, 2001)

Repeatability (intra assay variation) In duplicates

Statistical methods for results significance Fisher’s PLSD

Software (source, version) Microsoft Excel 2003 Data Analysis

B. qPCR information for ChIP assay

**Animals**

Description *Xenopus laevis* 1 years old (from Watanabe Breeding, Hyogo, Japan)

**Experimental design**

Definition of experimental and control groups Fed group: Fed 8 crickets/frog every day (22 days).

Fased group: Fasted in experiment term (22 days).

Re-fed group: Fasted 21 days and fed 8 crickets/frog only last day.

Number within each group n = 8

**Nucleic acid extraction**

Procedure and/or instrumentation Phenol-chloroform extraction

Name of kit and details of any modifications No kits were used

**qPCR target information**

Gene symbol See the text and Fig. S2

Sequence accession number See Table S2

Location of amplicon See Table S2

Amplicon length See Table S2

In silico specificity screen (BLAST, and so on) The primer specificity was confirmed using BLAST search.

**PCR oligonucleotides**

Primer sequences See Table S2

**qPCR protocol**

Complete reaction conditions Final volume of reaction regents:

12.5 μL 2 x Power SYBR Green Master Mix,

2 μL 2.5 μM each Primer,

8.5 μL Diethylpyrocarbonate-treated water,

2 μL precipitated DNA sample

Reaction volume and amount of cDNA/DNA Reaction volume, 25 μL

Primer, (probe), Mg^2+^, and dNTP concentrations Each 200 nM (final conc.)

Power SYBR Green Master Mix includes Mg^2+^ and dNTP, whose concentrations are unclear.

Polymerase identity and concentration Power SYBR Green Master Mix includes AmpliTaq Gold DNA Polymerase, whose concentration is unclear.

Buffer/kit identity and manufacturer Power SYBR Green Master Mix,

Applied Biosystems (Foster City, CA, USA), Cat. No. 4367659

Exact chemical composition of the buffer Unclear

Additives (SYBR Green I, DMSO, and so forth) Power SYBR Green Master Mix includes SYBR Green I and Passive reference, whose concentrations are unclear.

Manufacturer of plates/tubes and catalog number ABgene PCR Detection plates (Thermo Scientific, Yokohama, Japan), Cat. No : AB-1100

Complete thermocycling parameters 95°C for 10 min, and 40 cycles of 95°C for 15 s, 60°C for 1 min, and 50°C for 2 min

Manufacturer of qPCR instrument ABI Prism 7000 Sequence Detection System

(Applied Biosystems, Foster City, CA, USA)

**Data analysis**

qPCR analysis program (source, version) ABI PRISM 7000 SDS software version 1.0 (build 81 rev3)

Method of Cq determination Following the instruction manual, Cqs were arbitrarily determined the appropriate position.

Outlier identification and disposition None

Description of normalization method The formula 2^-Cq^. The Cq values of the ChIP signals were expressed as percentages of the ChIP signals for the input DNA.

Statistical methods for results significance Fisher’s PLSD

Software (source, version) Microsoft Excel 2003 Data Analysis

Chomczynski P, Sacchi N. (1987) Single-step method of RNA isolation by acid guanidinium thiocyanate-phenol-chloroform extraction. Anal Biochem. 162, 156-9.

Livak KJ, Schmittgen TD. (2001) Analysis of relative gene expression data using real-time quantitative PCR and the 2^-ΔΔCt^ Method. Methods. 25, 402-8.
